# Light-Regulation of Tryptophan Synthase by Combining Protein Design and Enzymology

**DOI:** 10.3390/ijms20205106

**Published:** 2019-10-15

**Authors:** Andrea C. Kneuttinger, Stefanie Zwisele, Kristina Straub, Astrid Bruckmann, Florian Busch, Thomas Kinateder, Barbara Gaim, Vicki H. Wysocki, Rainer Merkl, Reinhard Sterner

**Affiliations:** 1 Institute of Biophysics and Physical Biochemistry, University of Regensburg, Universitätsstraße 31, 93053 Regensburg, Germany; Andrea.Kneuttinger@ur.de (A.C.K.); Stefanie.Zwisele@ur.de (S.Z.); Kristina.Straub@ur.de (K.S.); Thomas.Kinateder@ur.de (T.K.); Barbara.Gaim@ur.de (B.G.); Rainer.Merkl@ur.de (R.M.); 2 Institute of Biochemistry, Genetics and Microbiology, University of Regensburg, Universitätsstraße 31, 93053 Regensburg, Germany; Astrid.Bruckmann@ur.de; 3 Department of Chemistry and Biochemistry and Resource for Native Mass Spectrometry Guided Structural Biology, The Ohio State University, Columbus, OH 43210, USA; busch.151@osu.edu (F.B.); wysocki.11@osu.edu (V.H.W.)

**Keywords:** allostery, biocatalysis, enzymology, photo-control, protein design, unnatural amino acids

## Abstract

The spatiotemporal control of enzymes by light is of growing importance for industrial biocatalysis. Within this context, the photo-control of allosteric interactions in enzyme complexes, common to practically all metabolic pathways, is particularly relevant. A prominent example of a metabolic complex with a high application potential is tryptophan synthase from *Salmonella typhimurium* (TS), in which the constituting TrpA and TrpB subunits mutually stimulate each other via a sophisticated allosteric network. To control TS allostery with light, we incorporated the unnatural amino acid *o*-nitrobenzyl-*O*-tyrosine (ONBY) at seven strategic positions of TrpA and TrpB. Initial screening experiments showed that ONBY in position 58 of TrpA (aL58ONBY) inhibits TS activity most effectively. Upon UV irradiation, ONBY decages to tyrosine, largely restoring the capacity of TS. Biochemical characterization, extensive steady-state enzyme kinetics, and titration studies uncovered the impact of aL58ONBY on the activities of TrpA and TrpB and identified reaction conditions under which the influence of ONBY decaging on allostery reaches its full potential. By applying those optimal conditions, we succeeded to directly light-activate TS(aL58ONBY) by a factor of ~100. Our findings show that rational protein design with a photo-sensitive unnatural amino acid combined with extensive enzymology is a powerful tool to fine-tune allosteric light-activation of a central metabolic enzyme complex.

## 1. Introduction

The significance of enzymes for the production of highly pure compounds has increased in recent years because such biocatalysts possess many advantages over current synthetic processes, such as functionality at ambient temperature [1]. Nevertheless, wild-type enzymes often are not stable or active enough under harsh reaction conditions, requiring tailoring for their application as industrial biocatalysts. Within this context, protein engineering approaches and specifically directed evolution made it possible to alter and improve enzyme function toward defined synthetic goals [2,3,4,5]. Beyond this step, however, enzymes still need to be assembled in cascades, spatially organized, and put under temporal control to precisely modulate the process outcome [1,5].

Light is very well-suited for this purpose. It is noninvasive, offers high-resolution in space, time, and intensity, and hence allows for sensitive tuning of enzyme activity [6,7]. Various strategies have been established that range from genetic modification with light-responsive photoreceptors in the field of optogenetics [8,9] to chemical functionalization with small molecular ligands [7,10]. Here, we concentrated on light-regulation of enzymes by means of unnatural amino acids (UAAs). This sophisticated approach to design light-regulated proteins has been well-investigated and implemented, especially in in vivo studies [11,12]. UAAs can be incorporated in response to an amber stop codon using an evolved, orthogonal aminoacyl-tRNA synthetase (aaRS)/tRNA pair [13]. Thus, an advantage of UAAs, especially over small molecular ligands, is that every enzyme molecule carries this light-responsive unit independent of dissociation rates.

One of the earliest examples and a very frequently used light-sensitive UAA is *o*-nitrobenzyl-*O*-tyrosine (ONBY) [14]. ONBY belongs to the class of caged UAAs that bear an additional protecting group on a natural amino acid scaffold [11,12]—which, in the case of ONBY, is a tyrosine. By introducing caged UAAs into essential positions, enzymes can be deactivated. Irradiation with light then releases the protecting group in a so-called decaging reaction and recovers protein activity. In the last decade, ONBY has been used to successfully light-regulate enzymes, such as β-galactosidase [14] or DNA and T7 RNA polymerase [15,16], other proteins, such as in cell-signaling networks [17], and even pro-drugs, such as anthrax toxin lethal factor, which led to photo-controlled therapy of cancer cells in mice [17]. In most applications of caged UAAs, enzyme activity could be light-activated by a factor of ~10 [17,18,19,20]. For temporal control of enzymes in industrial biocatalysis, however, higher light activation factors (LAFs) are desirable.

Here, we addressed this goal by considering two additional aspects to recent studies. First, the introduction of ONBY and other caged UAAs has mostly been restricted to the active site of enzymes. However, many enzymes are more complex and are allosterically regulated at sites outside the catalytic center in practically all metabolic pathways [21,22]. In particular, allostery in multi-enzyme complexes describes the activation of one subunit by binding of a ligand to another subunit [23]. Thus, light-regulation of allostery becomes most interesting for the temporal control of synthetic processes in enzyme cascades that generally mimic metabolic pathways [1]. Second, previously designed photo-controllable enzymes have been rarely studied in biochemical detail in order to understand how the UAA affects enzyme activity. Nonetheless, this knowledge might allow for the fine-tuning and optimization of the light activation process.

**Figure 1:**
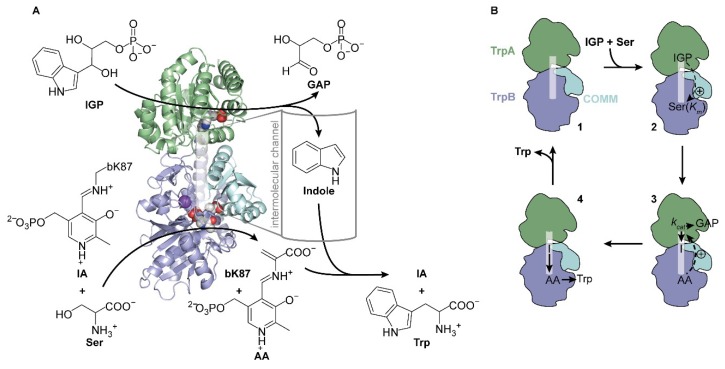
Scheme of the structure and function of tryptophan synthase (TS). (**A**) Reaction and crystal structure (PDB ID: 1a5s) of TS from *S. typhimurium*. TrpB (blue) catalyzes the condensation of serine (Ser) and indole to tryptophan (Trp) in the presence of the cofactor pyridoxal phosphate (PLP), which exists as internal aldimine (IA) bound to bK87 in TrpB. Serine initially condenses with IA to the aminoacrylate (AA), which further reacts with indole that is produced by TrpA (green) from indole-3-glycerol phosphate (IGP) and that travels through an intermolecular channel (transparent white) to the active site of TrpB. The active site of TrpA is marked by an IGP analogue, the active site of TrpB is marked by AA and a sodium cation, and the allosterically essential COMM domain is shown in cyan. (**B**) The activity of TS is tightly controlled by the presence of both substrates, IGP and serine, and by rearrangements of the communication domain (COMM). Starting from the apo-state (**1**), IGP positively stimulates binding of serine in TrpB, resulting in a lower *K_m_^Ser^* value (**2**), while binding of serine and formation of AA leads to an increase of TrpA activity as manifested in a higher *k_cat_* value (**3**). After indole (I) channeling, reaction with AA, and subsequent release of tryptophan (**4**), the apo-state is regenerated (**1**).

To test whether high LAFs can be reached by implementing these two aspects, we chose tryptophan synthase (TS) from *Salmonella typhimurium* as a model enzyme relevant for pharmaceutical biocatalysis [24]. TS is a multi-enzyme complex with a sophisticated allosteric machinery that catalyzes the biosynthesis of the essential amino acid tryptophan [25,26,27]. The heterotetrameric complex is comprised of two monomeric TrpA subunits that enclose one homodimeric TrpB subunit, resulting in an αββα quaternary structure [28]. The functional unit of TS consists simply of one TrpA and one TrpB molecule (Figure 1A). TrpA catalyzes the retro-aldol reaction of indole-3-glycerol phosphate (IGP) to glyceraldehyde-3-phosphate (GAP) and indole, which travels through an ~25 Å-long intermolecular channel to the active site of TrpB [29,30]. In TrpB, indole reacts with serine in a pyridoxal phosphate (PLP) catalyzed condensation reaction. The reaction product of serine and PLP, which is initially bound to a lysine residue of TrpB (bK87; b for TrpB) as an internal aldimine (IA), is an aminoacrylate (AA) intermediate [31]. Channeled indole reacts with AA at the TrpB active site to form tryptophan and regenerate IA.

TS is allosterically regulated in a bidirectional manner (Figure 1B) [26]. The binding of IGP in TrpA lowers the *K_m_* for serine in TrpB [32], and the subsequent formation of AA induces a higher *k_cat_* of the IGP lyase TrpA reaction [33]. Allosteric communication between both subunits is mainly mediated by structural rearrangements of the communication domain (COMM; bG102–bG189) in TrpB [34]. Moreover, monovalent cations can alter the catalytic efficiency of TS depending on their ion radius. Large cations, such as cesium, enhance the formation of AA [35], while small cations, such as sodium and potassium, facilitate the reaction of AA with indole [36]. Most importantly, both subunits only show residual activity as isolated monomer (TrpA) and homodimer (TrpB), respectively, and are significantly activated by the complexation event [37]. Frances Arnold and coworkers could circumvent this allosteric regulation by creating a standalone TrpB subunit that was highly active even without its partner protein [38,39]. Furthermore, the same researchers and another group developed TS and standalone TrpB variants that were able to synthesize various tryptophan derivatives such as 4-nitrotryptophan [24], a precursor compound relevant for agrochemistry [40] and pharmacology [41], pharmacologically relevant β-alkyl tryptophan [42,43], and tryptophan products with a new stereocenter at the γ-position [44].

In this study, we used the rational protein design approach of caged UAAs for the creation of light-dependent enzymes to activate enzyme allostery in TS from *S. typhimurium*. For this purpose, we incorporated the UAA ONBY in the intermolecular channel, close to or in the COMM domain, and at the interface of TrpA and TrpB. As a result, we initially identified an ONBY-TrpA variant, in which the TrpA, TrpB, and TS reactions were light-activated >5-fold. In a proceeding study of enzyme kinetic and biochemical characteristics of TS, we observed that ONBY influences both substrate binding and turnover and directly interferes with the allosteric activation of both subunits. Ultimately, we used optimized reaction conditions based on these observations to light-activate TS with a factor of ~100.

## 2. Results and Discussion

### 2.1. Kinetic Characterization of TS from Salmonella typhimurium

Tryptophan synthases from several organisms are well-established as models for understanding enzyme function-structure relationship. Most functional studies have been performed with tryptophan synthase from *Escherichia coli* (*ec*TS), while most structural studies utilized tryptophan synthase from *S. typhimurium* (TS, for the sake of simplicity). Thus, we decided to use TS for the in silico selection of positions and the subsequent experimental investigation of ONBY-variants.

Before we started the rational design of light-activatable TS, however, we kinetically characterized it in comparison to *ec*TS. The turnover of IGP by TrpA can be continuously followed spectrophotometrically in a well-established coupled assay with GAP dehydrogenase [45]. In contrast, the detection of tryptophan produced by TrpB has so far been limited to a problematic spectrophotometric assay, which uses a wavelength of 290 nm where a strong background absorbance stems from the enzyme itself [46], or by a complex mass spectrophotometric approach [47]. However, a more reliable spectrophotometric assay for the detection of tryptophan coupled to the tryptophan oxidase VioA and the peroxidase from horseradish (HRP) was recently developed [48], which we adapted for our kinetic studies of TrpB (Figure S1).

Steady-state kinetics were performed for the TrpA and TrpB partial reactions, as well as the overall TS reaction with varying concentrations of one substrate and a constant saturating concentration of the other substrates (Figure S2). The values for the steady-state kinetic constants, which were determined by fitting the data with the Michaelis-Menten equation, are shown in Table 1. All kinetic data of TS and *ec*TS were very similar, except for *K_m_^indole^*, which was ~5-fold higher for TS than for *ec*TS. Consistent with the recorded allosteric mechanism [32,33], *K_m_^Ser^* was ~2-fold reduced if IGP was used as substrate in the TS reaction instead of indole in the TrpB reaction, though the *k_cat_* of TrpB stayed the same.

We compared our results for *ec*TS with published data for the same enzyme [49]. Although the activity of TrpA and values for *K_m_^indole^* and *K_m_^Ser^* are very similar, we noticed that our *k_cat_* of the overall TS reaction is 3-fold lower and that our *K_m_^IGP^* is up to 8-fold lower than the reported values (*k_cat_* = 84 min^−1^; *K_m_^IGP^* = 140 µM for TrpA and *K_m_^IGP^* = 69 µM for TS). However, the TS reaction was previously followed using the GAP dehydrogenase assay [45,49], as the TrpA reaction was assumed to be rate limiting [50], and reactions were started with IGP instead of TS. By pursuing the same protocol, we obtained a *k_cat_* value (57 min^−1^ for *ec*TS) and a *K_m_^IGP^* (68 µM for *ec*TS) that are comparable to the published data, explaining this variation. Moreover, we observed a 23-fold deviation of the TrpB activity. As already indicated, the previously used spectrophotometric assay at 290 nm is susceptible toward errors, as the majority of the absorbance signal is produced by the protein itself. We therefore trust that our data are robust, especially because we experimentally proved the reliability of the VioA assay (Figure S1B) and because all *k_cat_* values of the TrpB and TS reaction were alike (21–28 min^−1^).

Using the ability to distinguish between GAP production and tryptophan production with our newly established VioA assay, we were able to reliably compare TrpA and TrpB activity during the TS reaction. As mentioned above, the TrpA reaction was thought to be rate-limiting [50], and we could confirm that this was the case in the absence of serine. However, GAP production increased 38-fold in the presence of serine (TS: *k_cat_* = 51 min^−1^, *ec*TS *k_cat_* = 57 min^−1^) and therefore exceeded tryptophan production more than 2-fold (TS: *k_cat_* = 21 min^−1^, *ec*TS: *k_cat_* = 28 min^−1^).

### 2.2. Identification and Characterization of An Efficient Position for Light-Activation of TS

**Figure 2:**
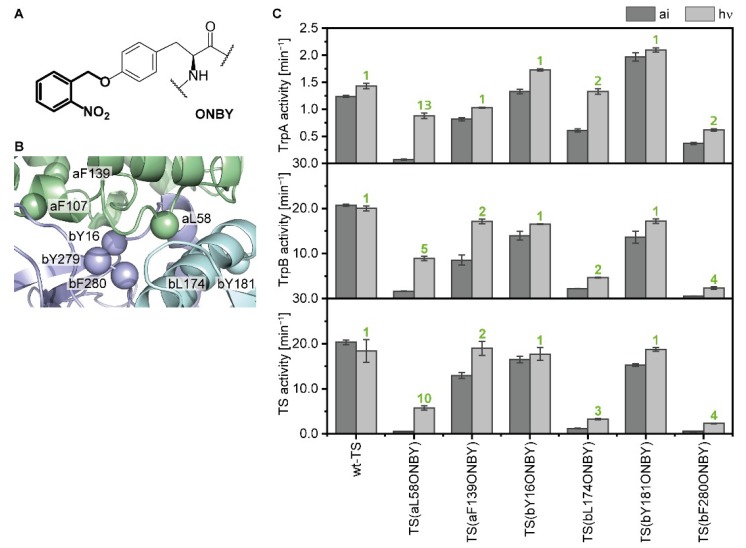
Identification of a light-responsive TS. (**A**) Chemical structure of the UAA *o*-nitrobenzyl-*O*-tyrosine (ONBY) carrying a photo-labile protecting group (bold). (**B**) Positions (spheres) of TrpA (green) and TrpB (blue; COMM domain in cyan) which were chosen for ONBY incorporation (PDB ID: 1a5s). (**C**) Activity screening of six expressed, purified, and mass spectrometry (MS)-confirmed ONBY-variants in complex with the wild-type partner protein. Activities of the TrpA, TrpB, and TS reaction were recorded of the proteins in their “as isolated” (ai) state and after irradiation with UV light (hν; 365 nm, 30 min) in substrate saturation (200 µM IGP, 500 µM indole, 5 mM serine) and compared to wild-type activities. LAFs were calculated as activity ratio V_hν_/V_ai_ and are shown above the bars (green). For reaction conditions, see Figure S2.

We chose eight positions in TS for the incorporation of ONBY (Figure 2A). The positions were localized in the intermolecular indole channel (bY279, bF280), close to or in the COMM domain (aL58, a for TrpA; bL174, bY181), and at the interface between TrpA and TrpB (aF107, aF139, bY16) (Figure 2B). In addition to tyrosine, we only chose phenylalanine and leucine residues, as they are most similar to tyrosine with respect to size and hydrophobicity. Seven of the eight ONBY-variants could be expressed in *E. coli* cells with the help of the previously designed aaRS/tRNA pair [14] and were purified with high yields of 10–185 mg per liter expression medium. ONBY incorporation was analyzed by tryptic digest coupled with mass spectrometry (MS) analysis and the purity of the proteins was confirmed by SDS-PAGE (Figure S3). The aF107ONBY variant was discarded after this step because we could not detect ONBY in MS analysis. From now on, we use the term a/bposONBY to name TrpA or TrpB proteins containing ONBY at position pos, e.g., aF107ONBY designates the TrpA protein with ONBY at position 107. Likewise, TS(aF107ONBY) describes the TS complex of aF107ONBY with wild-type TrpB.

The remaining six ONBY-variants were screened for their ability to modify the TrpA, TrpB, and TS reaction in their “as isolated” (caged) states and after decaging with UV light at a wavelength of 365 nm (Figure 2C). TS(aF139ONBY), TS(bY16ONBY), and TS(bY181ONBY) showed wild-type-like activities and no significant activation by light, while TS(aL58ONBY), TS(bL174ONBY), and TS(bF280ONBY) reached LAFs of 2–13. We chose TS(aL58ONBY) for our further investigations, as it reached 30–70% wild-type activity in all reactions after irradiation, whereas TS(bL174ONBY) and TS(bF280ONBY) only reached 18% and 13% wild-type activity, respectively, in the TrpB and TS reactions after irradiation.

**Figure 3:**
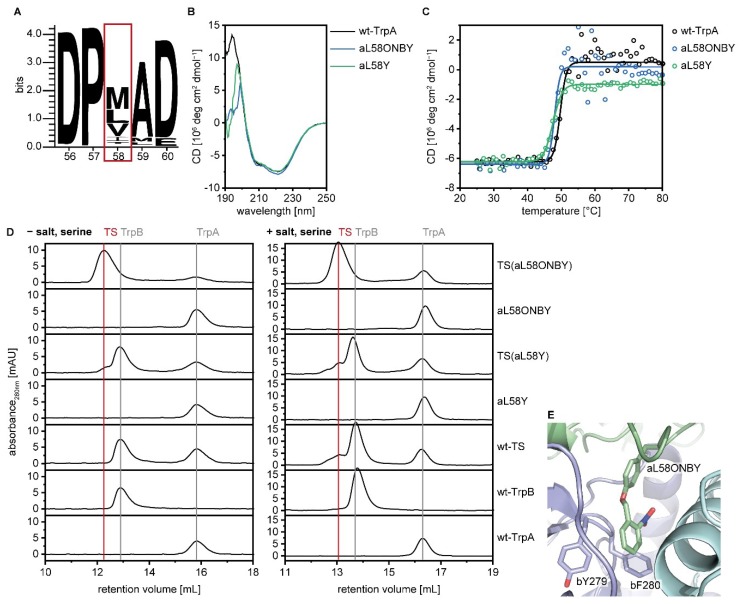
Biochemical characterization of wild-type TrpA, aL58ONBY, and aL58Y. (**A**) Sequence logo of positions 56–60 in TrpA. (**B**) Circular dichroism (CD) spectra of 10–15 µM wild-type TrpA, aL58ONBY, and aL58Y in 50 mM Tris·HCl pH 7.5. (**C**) CD thermal stability measurements recorded at 210 nm of wild-type TrpA (*T*_m_ = 50 °C), aL58ONBY (*T*_m_ = 48 °C), and aL58Y (*T*_m_ = 47 °C). (**D**) Analytical size-exclusion chromatography (Superdex S75 column) of the monomeric subunits and the TS complexes of wild-type TrpA, aL58ONBY, and aL58Y (30 µM) in 50 mM Tris·HCl pH 7.5 or (65 µM) in 50 mM Tris·HCl pH 7.5, 100 mM NaCl, 5 mM serine. (**E**) Positioning of the ONBY residue as determined in a molecular dynamics (MD) simulation after 100 ns of TS(aL58ONBY) (TrpA: Green, TrpB: Blue, COMM domain: Cyan). Representativeness of this snapshot has been verified as described in materials and methods.

Decaging of aL58ONBY leads to a point mutation in TrpA (L→Y). Hence, we expressed and purified aL58Y. TS(aL58Y) exhibited almost 100% wild-type TrpA activity and 50% wild-type TrpB and wild-type TS activities. It is not self-evident that a leucine to tyrosine mutation barely influences wild-type activity, however, a sequence logo of TrpA showed that for position 58 only the hydrophobic character is conserved (residues Met, Leu, Val, Ile, and Thr; Figure 3A), with which tyrosine complies. Circular dichroism (CD) spectroscopy further demonstrated that proteins aL58ONBY and aL58Y are both properly folded monomers and are thermally stable (Figure 3B,C). Remarkably, while wild-type TrpA and aL58Y only formed transient complexes with wild-type TrpB that were mainly detected in presence of salt and an excess of serine, aL58ONBY produced a highly stable complex in analytical size exclusion chromatography (Figure 3D).

Of our six tested ONBY-variants, the three in which the side chain points into the intermolecular indole channel (aL58ONBY, bL174ONBY, and bF280ONBY) showed a light-activation potential in TS. In order to explain this finding, we ran a molecular dynamics (MD) simulation of TS(aL58ONBY) based on the TS crystal structure with bound IGP-analogue in TrpA and aminoacrylate in TrpB (PDB ID: 1a5s). In this structural simulation, the side chain of ONBY positions itself in the channel and adopts a pinwheel arrangement with the two channel residues bY279 and bF280. Such a configuration has been shown to maximize the π–π-interactions in benzene trimers [51] and thus might explain the stabilized complex formation that we observed in size exclusion chromatography. Furthermore, bY279 and bF280 have been designated as gating residues of the channel, as they have been observed in positions that result in an either open [52] or closed [52,53] channel. The presence of ONBY close to this gate, at positions aL58, bL174, and bF280, likely leads to major perturbations of the allosteric communication, which we investigated further for aL58ONBY.

### 2.3. Inhibition of Catalytic Actvity of TS(aL58ONBY) Can Be Reversed by Light

Understanding how ONBY interferes with the different catalytic activities of TS is the basis from which we intend to derive reaction conditions that allow for most efficient light-activation of allostery. Hence, we measured all kinetic data of “as isolated”, caged TS(aL58ONBY^ai^), and irradiated, decaged TS(aL58ONBY^hν^), and compared them to the kinetic data of TS(aL58Y) as a positive control for the decaged state (Figure S4; Table 2).

The *k_cat_* values of TS(aL58ONBY^ai^) for the two partial and the overall TS reaction were activated 8–18-fold by light, similar to the LAFs of 5–13-fold that were observed in the activity screening (Figure 2). In addition, *K_m_* values were decreased 2–6-fold by light. (The exception was *K_m_^indole^*, which showed a 4-fold increase upon irradiation that went along with substrate inhibition whose mechanistic basis remained unexplored.) The catalytic efficiency *k_cat_/K_m_* was consequently activated 22–72-fold as a combination of both *k_cat_* and *K_m_* effects.

**Figure 4:**
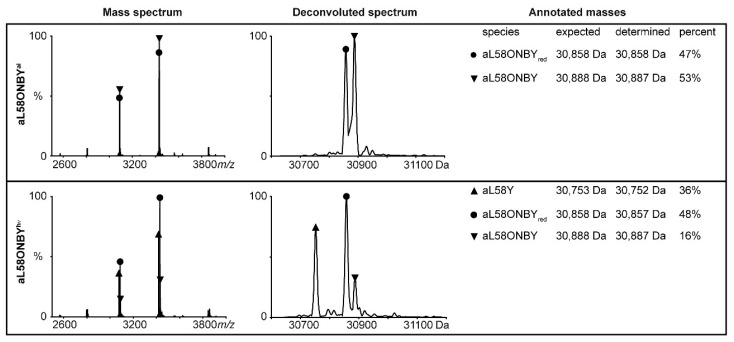
Native MS analysis of aL58ONBY^ai^ (upper panel) and aL58ONBY^hν^ (20 min irradiation at 365 nm; bottom panel). Three species—caged aL58ONBY, decaged aL58Y, and aL58ONBY reduced at the nitro group (“red”)—were detected.

The *K_m_* values of TS(aL58ONBY^hν^) matched the *K_m_* values of TS(L58Y) in most cases, while the *k_cat_* values only reached 49–74% of TS(aL58Y) activity. In native MS analysis, we confirmed that ~50% of TS(aL58ONBY) was not decageable, as the nitro group of ONBY, which is necessary for the photo-induction step [54], was reduced to an amine group (Figure 4, upper panel). The remaining intact TS(aL58ONBY) is, however, decageable to a large extent: A total of 36% decaged aL58Y were observed after 20 min of irradiation with UV-light whereas only 16% remained caged (Figure 4, bottom panel).

Taken together, TS(aL58ONBY^ai^) was less active than the positive control TS(aL58Y) with reduced *k_cat_* values, increased *K_m_* values, and consequently reduced *k_cat_/K_m_* values that were reactivated in TS(aL58ONBY^hν^) as a consequence of irradiation. For the direct photo-control of TS(aL58ONBY), we expect to reach high LAFs when we keep the substrate concentrations of IGP and serine below the respective *K_m_* in order to combine both *k_cat_* and *K_m_* effects. The potential could be further improved by extinguishing the problem of ONBY reduction by, for example, periplasmatic expression in an *E. coli* K12(DE3) strain [55].

### 2.4. Allosteric Activation of TrpA and TrpB is Inhibited by aL58ONBY

The described steady state kinetic analysis delivered the frame reaction conditions for the light-activation of TS(aL58ONBY). On this basis, we further analyzed the inhibitory effects of ONBY on the allosteric communication between TrpA and TrpB in order to grasp the full extent of this effect and to find further potential optimization conditions.

**Figure 5:**
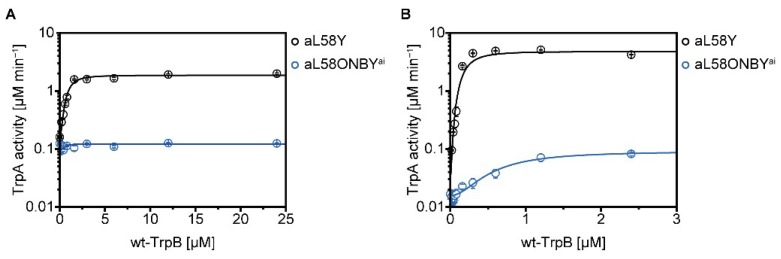
Activity titration experiments to show the stimulation of aL58ONBY^ai^ and aL58Y by wild-type TrpB in the absence (**A**) and presence (**B**) of serine. Reaction conditions: 50 mM Tris·HCl pH 7.8, 100 mM NaCl, 5 µM GAP dehydrogenase, 250 µM IGP, (5 mM serine), 5 mM NAD^+^, 20 mM Na_2_HAsO_4_, 40 µM PLP, 0–24(0–2.5) µM wild-type TrpB, and 1(0.2) µM TrpA. All reactions were performed in triplicates at 25 °C and data were fitted with the Hill equation (3) and a cooperativity factor of *H* = 2 representative of two TrpA monomers binding to one TrpB dimer.

The *k_cat_* value of TrpA was impaired 12-fold in TS(aL58ONBY^ai^) compared to the fully decaged TS(aL58Y) (Table 2), which indicates that allosteric activation of TrpA by complexation with TrpB might be affected by the caging group. To further test this conclusion, activity titration experiments were conducted in which the TrpA concentration was kept constant, while the wild-type TrpB concentration was gradually increased. We performed this experiment with both aL58ONBY^ai^ and aL58Y, first in the absence of serine (Figure 5A).

In fact, the TrpA activity of aL58ONBY^ai^ could not be increased by complexation with wild-type TrpB, while TrpA activity of aL58Y was stimulated 12-fold (Table 3).

Moreover, in TS(aL58Y), serine binding allosterically stimulated the *k_cat_* value of TrpA about 5-fold, whereas in TS(aL58ONBY^ai^), this activation was minimized to a factor of 1.5 (comparison of the TrpA and TS reactions in Table 2). Hence, the allosteric activation of TrpA caused by the binding of serine to TrpB was reduced in TS(aL58ONBY^ai^) compared to TS(aL58Y). In accordance with this finding, activity titrations in the presence of serine pronounced the effect described above: The TrpA activity of aL58ONBY^ai^ was only increased 4-fold by wild-type TrpB, while TrpA activity of aL58Y was stimulated 160-fold (Figure 5B, Table 3).

We concluded that the activating effect of wild-type TrpB was much weaker for aL58ONBY^ai^ compared to aL58Y and that this stimulation difference was increased by the presence of serine.

**Figure 6:**
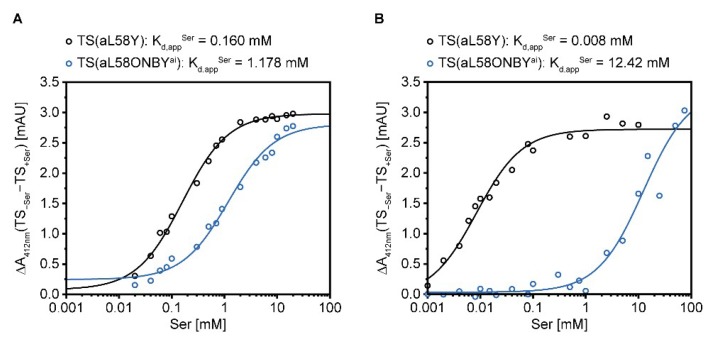
Absorbance titration experiments to show the influence of ONBY on binding of serine to TrpB in absence (**A**) or presence (**B**) of 100 mM cesium cations. The total change in TS absorbance at 412 nm, representative of the reduction in IA species, is plotted against the concentration of serine. The experimental data were fitted with the Hill equation (3) (*H* = 1).

Likewise, the *K_m_^Ser^* value of TrpB was not stimulated by the presence of IGP in TS(aL58ONBY^ai^), but was stimulated 9-fold in TS(aL58Y) (Table 2). This indicates that allosteric activation of TrpB by TrpA might be affected by the caging group. Absorbance titration experiments, in which the reduction of the IA signal at 412 nm was followed after the step-wise addition of serine, should clarify whether this effect is rooted in the covalent bond formation of serine and PLP or in later intermediate reaction steps of TrpB catalysis. We performed this experiment with TS(aL58ONBY**^ai^**) and TS(aL58Y), plotted the total change of the absorbance signal against the serine concentration, and estimated *K_d,app_^Ser^* values for covalent serine binding. For both TS(aL58ONBY**^ai^**) and TS(aL58Y) the *K_d,app_^Ser^* values, determined in absence of any cations (Figure 6A), were within the same scale of the *K_m_^Ser^* values for the TrpB reaction, determined in presence of 150 mM potassium ions (Table 2). Hence, the *K_m_^Ser^* differences between TS(aL58ONBY^ai^) and TS(aL58Y) primarily originated from the covalent binding event of serine.

Nevertheless, the *K_d,app_^Ser^* only differs by a factor of seven in TS(aL58ONBY**^ai^**) compared to TS(aL58Y). In order to test whether this difference can be enlarged by the use of a cation other than potassium, we repeated the absorbance titration experiment with cesium. As expected from wild-type studies [35], covalent serine binding as an indirect measure of AA formation was significantly stimulated (20-fold) in TS(aL58Y) (Figure 6B). However, in TS(aL58ONBY**^ai^**), cesium instead hampered (10-fold) this reaction. Consequently, the absolute difference of *K_d,app_^Ser^* in TS(aL58ONBY**^ai^**) and TS(aL58Y) in the presence of cesium was >1,000-fold.

Finally, we obtained a relatively clear impression of the inhibitory scope of ONBY on TS allostery. The suppression of three major allosteric activation events dominated TS(aL58ONBY) activity, namely the allosteric activation of TrpA by complexation with TrpB, the stimulation of AA formation in TrpB by IGP and cesium binding, and the activation of TrpA by serine binding. For the direct photo-control of TS(aL58ONBY), we could now further optimize the reaction conditions by keeping one partner protein in excess to ensure full activation by complexation after irradiation, and by adding cesium to enhance inhibition of covalent serine binding.

**Figure 7:**
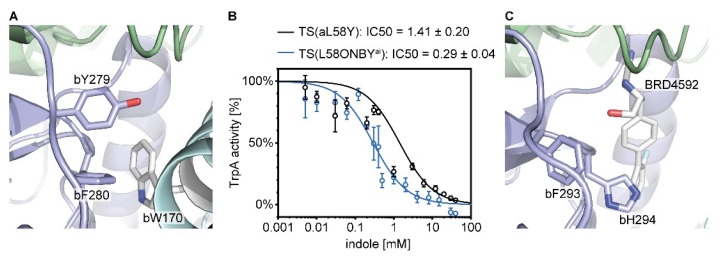
Blockade of the intermolecular indole channel by three different mechanisms. (**A**) The bC170 mutation to tryptophan (bW170) is located close to the two gating residues bY279 and bF280 (PDB ID 1fuy). (**B**) Product inhibition of TrpA in TS(aL58ONBY^ai^) compared to TS(L58Y) by increasing indole concentrations. Reaction conditions: 50 mM Tris·HCl pH 7.8, 100 mM NaCl, 5 µM GAP dehydrogenase, 55 µM IGP (~K_m_), 5 mM NAD^+^, 20 mM Na_2_HasO_4_, 40 µM PLP, 0–40 mM indole, and 1 µM TS (1:1 TrpA:TrpB). Data are shown ± SEM and were fitted with the dose-response equation (4). IC50 values are given ± SE. (**C**) TS inhibitor BRD4592 is bound in the intermolecular channel of *M. tuberculosis mt*TS close to the two gating residues bF293 (corresponds to bY179 in TS) and bH294 (corresponds to bF280 in TS) (PDB ID 5tci). TrpA: Green, TrpB: Blue, COMM: Cyan.

With regard to the suppression of TS allostery, we found parallels between the effect of the caging group in TS(aL58ONBY**^ai^**) and the mutation of bC170, lining the intermolecular channel, to a bulky tryptophan (Figure 7A) [56,57]. This mutation caused conformational changes of the gating residues bY279 and bF280, and unfavorable movements of the COMM domain [58] with consequences on the allosteric communication pathway [52,59] and structural changes in the TrpB active site that impaired stabilization of bound serine. As a result, activation of both the *k_cat_* value of TrpA [33] and the binding constant *K_d_^Ser^* of TrpB [58] were impaired 100-fold. Additionally, tryptophan bW170 obstructed the channel so that the rate constant for indole channeling was 5,000-fold reduced [57].

We therefore reasoned that indole channeling might also be blocked in TS(aL58ONBY**^ai^**). However, we chose a simpler, though indirect, measure of this effect. We assumed that indole might accumulate more strongly in TrpA when the channel is clogged and that this might lead to stronger product inhibition of TrpA by indole. Thus, we measured the dependence of TrpA activity on externally added indole and determined the IC50 value, which represents the indole concentration at which TrpA loses 50% of its activity (Figure 7B). In TS(aL58ONBY**^ai^**), the IC50 was 5-fold lower than in TS(aL58Y). This confirms that indole channeling was slowed down in TS(aL58ONBY**^ai^**), possibly by a trimeric π–π-interaction of ONBY with bY279 and bF280 (Figure 3E).

Clogging of the indole channel has also been shown recently for two allosteric inhibitors of TS from *Mycobacterium tuberculosis* that bind within the channel [47,60]. The inhibitory mechanism of one of them, BRD4592 (Figure 7C), was described in detail [47]. Similar to ONBY, it stabilizes the interaction between TrpA and TrpB, blocks the indole transport, and inhibits both TrpA and TrpB reactions. Nevertheless, the mode of inhibition differs as it increases instead of reducing substrate affinity, stabilizes instead of destabilizing catalytic TrpB intermediates, and, finally, increases tryptophan product inhibition. Hence, inhibition of TS by molecules that are centrally positioned within the indole channel is quite effective and versatile. In addition, the inhibitory effect of ONBY in TS(aL58ONBY**^ai^**) can be revoked upon irradiation with light.

### 2.5. Optimized Light-Activation of TS by Combination of Kinetic Effects

**Figure 8:**
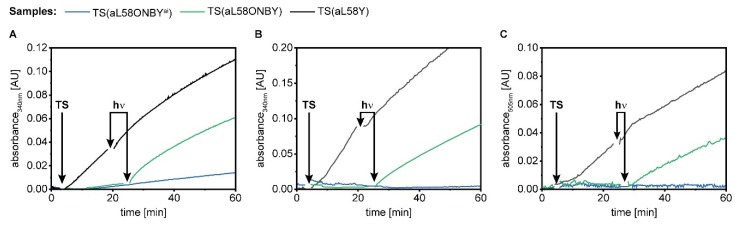
Direct light-activation of TS(aL58ONBY) compared to a TS(aL58ONBY^ai^) control (kept in the dark) and a TS(aL58Y) control (irradiated simultaneously). (**A**) TrpA reaction: 50 mM Tris·HCl pH 7.8, 100 mM NaCl, 5 µM GAP dehydrogenase, 100 µM IGP, 5 mM NAD^+^, 20 mM Na_2_HasO_4_, 40 µM PLP, and 1 µM TS (5:1 TrpA:TrpB). (**B**) TrpA reaction in presence of serine: 50 mM Tris·HCl pH 7.8, 100 mM NaCl, 5 µM GAP dehydrogenase, 250 µM IGP, 1 mM serine, 5 mM NAD^+^, 20 mM Na_2_HasO_4_, 40 µM PLP, and 0.2 µM TS (5:1 TrpA:TrpB). (**C**) TS reaction: 50 mM Tris·HCl pH 7.5, 100 mM CsCl, 250 mM IGP, 1 mM serine, 2 g/L VioA, 0.3 g/L HRP, 40 µM PLP, 1 mM phenol, 1 mM 4-aminoantipyrin, and 0.2 µM TS (5:1 TrpA:TrpB). All three reactions were started with the enzyme complex, followed for ~20 min, irradiated for 1.5 min at 365 nm while enzyme velocity was still in the initial linear range, and followed further up to 60 min.

We identified a light-activatable TS by rational protein design with the caged UAA ONBY and used classical enzymology to identify reaction conditions that maximize this effect. Now we tested these conditions gradually in three direct photo-control setups (Figure 8). For this, we followed TS(aL58ONBY) activity in two samples: One reaction assay was kept in the dark (“TS(aL58ONBY^ai^)”), while the other was irradiated during the initial linear activity phase of the enzyme (“TS(aL58ONBY)”). In order to keep the irradiation time to a minimum, we exchanged our conventional UV lamp for a high-power LED (365 nm) that allowed us to decage ONBY within 1.5 min [61]. Moreover, we applied the same treatment as control on a reaction assay following TS(aL58Y) activity (“TS(aL58Y)”).

In the first setup, we measured the TrpA reaction of TS(aL58ONBY) and used the observations that ONBY increases *K_m_^IGP^* and inhibits activation of TrpA by complexation with wild-type TrpB. Hence, we applied IGP concentrations below *K_m_^IGP^* and a 5-fold excess of TrpA over TrpB to ensure maximal activation after irradiation (Figure 8A). TrpA activity of TS(aL58ONBY) was light-activated 4-fold in this reaction (Table 4). Its initial velocity prior to irradiation *V_0_* was equivalent to the activity of the TS(aL58ONBY^ai^) control and the *k_cat_* of TrpA determined in steady state kinetics (Table 2). Its velocity after irradiation ***V_hν_*** was ~57% of the initial velocity of TS(aL58Y), which matched the amount of decageable enzyme quite well (Figure 4). However, TS(aL58Y) lost ~34% activity after irradiation and the LAF of 4 was lower than the value of 22 that was observed for the catalytic efficiency differences between TS(aL58ONBY^ai^) and TS(aL58ONBY^hν^) (Table 2).

In the next experiment, we added serine to the TrpA reaction of TS(aL58ONBY) at a concentration below *K_m_^Ser^* of TS(aL58ONBY) (Figure 8B). This change of reaction conditions was based on the observation that ONBY inhibits activation of TrpA by complexation with wild-type TrpB even stronger in the presence of serine. Hence, TrpA activity of TS(aL58ONBY) was light-activated 42-fold (Table 4), which matched quite well the value of 56 that was observed for the catalytic efficiency differences between TS(aL58ONBY^ai^) and TS(aL58ONBY^hν^) in the TS reaction (Table 2). Again, its initial velocity *V_0_* was equivalent to the activity of the TS(aL58ONBY^ai^) control and its velocity after irradiation achieved ~47% of initial TS(aL58Y) activity. TS(aL58Y) lost 44% activity after irradiation.

In the third and final experiment, we measured the TS reaction of TS(aL58ONBY) and used the same reaction conditions as for the TrpA reaction, but added cesium instead of potassium (Figure 8C). This modification was based on the observation that cesium hampered covalent binding of serine to the PLP cofactor in TS(aL58ONBY**^ai^**), but allowed tighter binding of serine in TS(aL58Y). Remarkably, the activity of both the TS(aL58ONBY**^ai^**) control and the TS(aL58ONBY) sample before irradiation sank below the detection limit of the plate reader (~10^−5^ AU min^−1^) which translates to a maximum activity of ~0.02 min^−1^, more than 10-fold less than in steady state kinetics (Table 2). As a result, TS(aL58ONBY) was light-activated >141-fold and reached ~57% of the initial TS(aL58Y) activity. TS(aL58Y), again, lost ~37% activity after irradiation.

These experiments summarize the success of our combined approach. We introduced the caged UAA ONBY at a position outside the active site and, in doing so, suppressed the allosteric machinery of the multienzyme complex TS. Furthermore, by investigating the inhibitory scope of ONBY, we identified reaction conditions that allowed the fine-tuning of the light activation effect. This finally led to enzyme activation of ~100-fold upon irradiation with light, whereas studies that applied caged UAAs simply in the active site of an enzyme only achieved an average LAF of 10 [17,18,19,20]. Factors of ~100 are far better suited for the temporal control of enzymes in biocatalysis [1], and our approach for TS might be applied for the photo-controlled production of tryptophan derivatives [24,42,43].

At a first glance, keeping substrate concentrations below the saturation level seems to be a disadvantage for industrial applications. However, industrially relevant compounds are often derived from hydrophobic substrates with low solubility in aqueous buffers and thus concentrations below *K_m_* are advisable. Moreover, enzyme cascades, which are relevant for biocatalysis [1], imitate metabolic biosynthesis pathways and substrate concentrations are thus not likely to reach saturation within the cascade, even if the first substrate is added in saturation.

Despite this success, we need to address some improvements in future studies. One major drawback is the high activity loss of ~40% in TS(aL58Y), which most likely indicates that the photo-sensitive PLP cofactor of TrpB was damaged by UV light. In all preceding experiments, we pre-irradiated only TrpA and hence only became aware of this effect during the direct light-activation experiments. This problem might be overcome using visible light photo-labile cages. These protecting groups are, however, still relatively rare [62], and have so far mainly been used to cage biologically active compounds, such as the neurotransmitter γ-aminobutyric acid [63]. Furthermore, reversible light-regulation is desirable. Light-switchable UAAs already exist [64,65,66], but in most studies, only light-regulation factors of 1.5–3 were achieved. Nevertheless, in our most recent study, we were able to obtain a light-regulation factor of ~10 by controlling the allosteric stimulation in the bi-enzyme complex imidazole glycerol phosphate synthase with the light-switchable UAA phenylalanine-4′-azobenzene (AzoF) [61]. However, at this point our strategy of photo-controlling allostery can only be intuitively applied to enzymes with an existing allosteric machinery. More design studies on allosteric and non-allosteric enzymes to attempt to induce allostery with light using caged or light-switchable UAAs will be necessary to grasp the full potential and allow reproducibility of this promising allosteric light-regulation approach.

## 4. Materials and Methods

### 4.1. Strains, Enzymes, and Chemicals

Expression strains used for production of TrpA, TrpB, *ec*TrpA, and *ec*TrpB, as well as of auxiliary enzymes VioA and GAP dehydrogenase, were purchased from Agilent Technologies [*E. coli* BL21 Gold (DE3), BL21-CodonPlus (DE3)-RIPL] (Santa Clara, CA, USA) and Novagen [*E. coli* BL21 (DE3) Rosetta] (Merck, Darmstadt, Germany). The following expression vectors ware taken from previously published work: pET24a_TrpB for TrpB expression [67], and pET28a_tmGAPDH for GAP dehydrogenase expression [68]. The plasmid pEVOL_ONBY for incorporation of ONBY into proteins was provided by Prof. Peter Schultz (Scripps Research Institute, La Jolla, CA, USA) [14,69]. HRP was purchased from Sigma Aldrich (St. Louis, MO, USA). IGP was produced enzymatically from 1-(*o*-carboxyphenylamino)-1-deoxyribulose-5-phosphate as described previously [70] and ONBY was purchased from Accela ChemBio (96% pure) (San Diego, CA, USA). All other chemicals were purchased from commercial sources and were of analytical grade or higher.

### 4.2. Subcloning of the trpA, ectrpA, ectrpB, and vioA Genes

The *trp*A gene was amplified from pBR322-st*trp*AB, provided by Prof. Ilme Schlichting, by PCR using the oligonucleotides 5′-AGC CAT ATG GAA CGC TAC GAA AAT TTA-3′ and 5′-GTG GTG CAA GCT TAT GCG CGG CTG GCG GCT TTC-3′. The amplification product was inserted into pET28a(+) at the *NdeI/HindIII* restriction sites. The *ectrp*A gene was amplified from *E. coli* MG1655 genomic DNA using the primers 5′-TAT ACA TAT GGA ACG CTA CGA A-3′ and 5′-GTG GTG CTC GAG ACT GCG CGT CGC CGC-3′ and inserted into pET21a(+) at the *NdeI/XhoI* restriction sites. The *ectrp*B gene was amplified from *E. coli* MG1655 genomic DNA using the primers 5′-TAT ACA TAT GAC AAC ATT ACT T-3′ and 5′-GTG GTG CTC GAG GAT TTC CCC TCG TGC-3′ and inserted into pET21a(+) at the *NdeI/XhoI* restriction sites. The *vio*A gene from *Chromobacterium violaceum* was purchased with *BsaI* cloning sites from GeneArt and inserted into pET28a_*Bsa*I [71] using Golden Gate Cloning [72]. All cloning products were checked by Sanger Sequencing (Microsynth Seqlab, Göttingen, Germany).

### 4.3. Site-Directed Mutagenesis of trpA and trpB

Stop codon point mutations (TAG, underlined) were introduced into pET28a_TrpA and pET24a_TrpB according to the protocol of the Phusion™ site-directed mutagenesis kit from Finnzymes (Thermo Fisher Scientific, Waltham, MA, USA) with 5′ phosphorylated and HPLC-purified primers (Metabion): 5′-GCC CTA CCA TCC AGA ATG CGA ACT TAC-3′ and 5′-CAT CGG CCT ACG GAT CGG AGA AG-3′ for aL58ONBY, 5′-GAA TCT GGT GTA GAA TAA CGG CAT AGA TG-3′ and 5′-GCG TAC ATT AGC AGG CCA ATC G-3′ for aF107ONBY, 5′-CCT AGC GCC AGG CAG CGT TAC G-3′ and 5′-GGG CCG ATT CTT CAA CCG GGA C-3′ for aF139ONBY, 5′-GGC GGC ATG TAG GTG CCG C-3′ and 5′-GAA TTC ACC AAA GTA GGG GTT GAG-3′ for bY16ONBY, 5′-TAG CGC GAC TGG TCC GGT AGT TAC-3′ and 5′-CGC CTC GTT ACA GGC ATC TTT TAG CG-3′ for bL174ONBY, 5′-CTG GTC CGG TAG TTA GGA AAC CG-3′ and 5′-TCG CGC AGC GCC TCG TTA C-3′ for bY181ONBY, 5′-GCG TTG GCA TCT AGT TCG GGA TG-3′ and 5′-GAC CAT GTT TAA GCG GCG GG-3′ for bY279ONBY, 5′-GCA TCT ATT AGG GGA TGA AAG CGC C-3′ and 5′-CAA CGC GAC CAT GTT TAA GCG G-3′ for bF280ONBY. Correct mutagenesis was checked by Sanger Sequencing (Microsynth Seqlab, Göttingen, Germany).

### 4.4. Expression and Purification of TS

The TrpA, TrpB, *ec*TrpA, and *ec*TrpB proteins were produced by heterologous gene expression in *E. coli* BL21 (DE3) Rosetta (TrpA), and BL21 Gold (DE3) (TrpB, *ec*TrpA, *ec*TrpB). Strains containing the respective vector were grown in 4 L lysogeny broth (LB) medium with kanamycin (TrpA, TrpB), ampicillin (*ec*TrpA), or ampicillin and 20 µM PLP (*ec*TrpB) at 37 °C to an OD_600_ of 0.6. Protein expression was induced with 0.5 mM IPTG. Incubation overnight at 20 °C was followed by harvesting of bacterial pellets and suspension in either 50 mM Tris·HCl pH 7.5, 150 mM NaCl, and 10 mM imidazole (TrpA, TrpB) or 100 mM KP pH 7.5, 300 mM KCl, and 10 mM imidazole (*ec*TrpA, *ec*TrpB). Proteins were obtained from the supernatant after sonication and repeated centrifugation steps, subjected to nickel-affinity chromatography (HisTrap™ FF Crude column, 5 mL, GE Healthcare), and eluted with a linear gradient of imidazole (10 mM→1 M TrpA, 10→750 mM TrpB, 10→500 mM *ec*TrpA and *ec*TrpB). Fractions containing the proteins were identified by SDS-PAGE analysis, pooled and further purified with a size-exclusion chromatography column (Superdex 75 HiLoad 26600, GE Healthcare, Chicago, IL, USA) by using 50 mM Tris·HCl pH 7.5 (TrpA, TrpB) or 100 mM KP pH 7.5, and 300 mM KCl (*ec*TrpA, *ec*TrpB) as running buffer. Fractions were checked on SDS-PAGE analysis for >90% purity, pooled, concentrated, and dripped into liquid nitrogen for storage at −80 °C.

For the incorporation of ONBY into TrpA and TrpB, a slightly adjusted expression protocol was used. After co-transformation of the expression vectors, carrying the desired TAG codon, and pEVOL_ONBY into *E. coli* BL21 Gold (DE3), the strains were grown in LB medium (6 L) at 37 °C to an OD_600_ of 0.6. Then, bacterial pellets were harvested by centrifugation at room temperature and suspended in terrific broth (TB) medium (600 mL). Bacterial growth was resumed to an OD_600_ of approximately 10 at 37 °C and incorporation was induced by addition of 1 mM ONBY and 0.02% l-arabinose. The omission of IPTG turned out to be favorable for expression, as higher yields could be obtained in its absence. The cultures were incubated overnight at 30 °C and proteins were purified as described above.

### 4.5. Expression and Purification of Auxiliary Enzymes

GAP dehydrogenase from *Thermotoga maritima* and VioA from *C. violaceum* were expressed in *E. coli* BL21-CodonPlus (DE3)-RIPL and BL21 Gold (DE3), respectively. Strains containing the respective vector were grown in 4 L lysogeny broth (LB) medium supplemented with kanamycin at 37 °C to an OD_600_ of 0.6. Protein expression was induced with 0.5 mM IPTG. Incubation overnight at 37 °C (GAP dehydrogenase) and 20 °C (VioA) was followed by harvesting of bacterial pellets and suspension in either 10 mM KP pH 7.5, 300 mM KCl, and 10 mM imidazole (GAP dehydrogenase) or 20 mM Tris·HCl pH 8.0, 300 mM NaCl, and 20 mM imidazole (VioA). Proteins were obtained from the supernatant after sonication and repeated centrifugation steps. For GAP dehydrogenase, the majority of *E. coli* proteins were precipitated in a heat step (20 min, 75 °C) and subsequent centrifugation. Proteins were subjected to nickel-affinity chromatography (HisTrap™ FF Crude column, 5 mL, GE Healthcare, Chicago, IL, USA) and eluted with a linear gradient of imidazole (10→500 mM). Fractions containing the proteins were identified by SDS-PAGE analysis, pooled and either dialyzed into 10 mM KP pH 7.5 (GAP dehydrogenase) or further purified with a size-exclusion chromatography column (Superdex 75 HiLoad 26/600, GE Healthcare, Chicago, IL, USA) using 20 mM Tris·HCl pH 8.0 as running buffer. The proteins were concentrated, and dripped into liquid nitrogen for storage at −80 °C.

### 4.6. Activity Measurements

In general, activity measurements were performed at 25 °C with a microplate reader (Infinite M200 Pro, TECAN, Männedorf, Switzerland) and activities were deduced from the initial slopes of the transition curves. The exact reaction conditions are given under each figure describing the respective experiment. IGP turnover by TrpA was determined continuously in a coupled enzymatic assay with GAP dehydrogenase as auxiliary enzyme and NAD^+^ as co-substrate. Activity was detected at *λ* = 340 nm according to NAD^+^ turnover [Δε_340_(NADH−NAD^+^) = 6300 M^−1^ cm^−1^]. The IGP- or indole-dependent tryptophan production by TrpB was followed continuously in a coupled, colorimetric enzymatic assay with VioA and HRP as auxiliary enzymes. Flavin-dependent VioA oxidized tryptophan producing the side-product peroxide, which again was turned over by HRP with colorless 4-aminoantipyrine and phenol to colored quinone imine. Activity was detected at *λ* = 505 nm according to formation of quinone imine [Δε_505_(quinone imine) = 6,400 M^−1^ cm^−1^]. All reactions were started by addition of the TS complex.

Caged ONBY-proteins were either used “as isolated” or irradiated for 30 min at 365 nm (conventional UV lamp, two fluorescent black light bulbs with 8 W, Sylvania, Erlangen, Germany; settings: 250 mA and 220 V). 30 µM of proteins were cooled in a metal rack during irradiation that was positioned in ~1 cm distance to the light source. ONBY-proteins were then mixed with the respective partner protein to form the TS complex and kept in the dark. Direct photo-control experiments were performed with the help of a 365 nm high-power LED (High Power LED, LED Engin, Osram, München, Germany; settings: 700 mA and 16 V) for 1.5 min.

### 4.7. Tryptic Digest and MS Analysis of Protein Sequences

Recombinant *S. typhimurium* TrpA and TrpB proteins were run on a 15% SDS-PAGE and stained with Coomassie G250 (SimplyBlue SafeStain, Lifetechnologies, Thermo Fisher Scientific, Waltham, MA, USA). Protein bands were cut out from the gel, washed with 50 mM NH_4_HCO_3_, 50 mM NH_4_HCO_3_/acetonitrile (3/1), 50 mM NH_4_HCO_3_/acetonitrile (1/1), and lyophilized. After a reduction/alkylation treatment and additional washing steps, proteins were *in gel* digested with trypsin (Trypsin Gold, mass spectrometry grade, Promega, Mannheim, Germany) overnight at 37 °C. The resulting peptides were sequentially extracted with 50 mM NH_4_HCO_3_ and 50 mM NH_4_HCO_3_ in 50% acetonitrile. After lyophilization, peptides were reconstituted in 20 µL 1% TFA and separated by reversed-phase chromatography. An UltiMate 3000 RSLCnano System (Thermo Fisher Scientific, Waltham, MA, USA) equipped with a C18 Acclaim Pepmap100 preconcentration column (100 µm i.d. × 20 mm, Thermo Fisher Scientific) and an Acclaim Pepmap100 C18 nano column (75 µm i.d. × 250 mm, Thermo Fisher Scientific, Waltham, MA, USA) was operated at a flow rate of 300 nL/min and a 60 min linear gradient of 4% to 40% acetonitrile in 0.1% formic acid. The LC was online-coupled to a maXis plus UHR-QTOF System (Bruker Daltonics, Billerica, MA, USA) via a CaptiveSpray nanoflow electrospray source. Acquisition of the MS/MS spectra after CID fragmentation was performed in data-dependent mode at a resolution of 60,000. The precursor scan rate was 2 Hz processing a mass range between *m*/*z* = 175 and *m*/*z* = 2000. A dynamic method with a fixed cycle time of 3 s was applied via the Compass 1.7 acquisition and processing software (Bruker Daltonics, Billerica, MA, USA).

Prior to database searching with Protein Scape 3.1.3 (Bruker Daltonics, Billerica, MA, USA) connected to Mascot 2.5.1 (Matrix Science), raw data were processed in Data Analysis 4.2 (Bruker Daltonics, Billerica, MA, USA). A customized database comprising the *S. typhimurium* entries from UniProt, as well as manually added sequences of the mutated TrpA and TrpB proteins and common contaminants, were used for a database search with the following parameters: Enzyme specificity trypsin with two missed cleavages allowed, precursor tolerance 10 ppm, MS/MS tolerance 0.04 Da. General variable modifications included in the search were deamidation of asparagine and glutamine, oxidation of methionine, carbamidomethylation, or propionamide modification of cysteine. A specific variable modification for identification of *o*-nitrobenzyltyrosine (ONBY) was also included. ONBY was detected as a modification of tyrosine, with each position of ONBY incorporation changed to a tyrosine in the query, respectively. Spectra of peptides containing ONBY were inspected manually to confirm ONBY incorporation and location.

### 4.8. MD Simulation

The MD simulations of TS(aL58ONBY) were conducted with Yasara [73], version 17.4.17, force field AMBER03 on the basis of the TS complex (PDB ID 1a5s, chains A, B). ONBY was incorporated into TrpA at position L58 and sterically oriented using a previously generated rotamer library [61]. A hexagonal simulation cell was created, which was 5 Å larger than the protein along each axis, filled with water to a density of 0.997 g/mL and with counterions to a final concentration of 0.9% NaCl. One simulation run of 100 ns length was performed with an initial equilibration step of 14 ps length for minimization. During the simulation run, snapshots were recorded every 20 ps, representing 5000 recorded steps. Of each snapshot, RMSD values were calculated on the initial and end structure proving convergence toward the end structure.

### 4.9. Generating a Sequence Logo for TrpA Subunits

Sequences of TrpA subunits were retrieved from InterPro family IPR002028 (version as of 20.05.2019,) [74]. This set of 29,540 sequences was aligned using *mafft* with the option *auto*. In order to generate a compact alignment, sequences solely responsible for inserts were removed. The remaining 19,683 sequences were realigned, and a sequence logo was built using weblogo3 [75] with default parameters.

### 4.10. Native MS Analysis

Identity of aL58ONBY and its decaging efficiency were analyzed by online buffer exchange MS using an UltiMate™ 3000 RSLC (Thermo Fisher ScientificWaltham, MA, USA) coupled to an Exactive Plus EMR Orbitrap instrument (Thermo Fisher ScientificWaltham, MA, USA) modified to incorporate a quadrupole mass filter and allow for surface-induced dissociation [76]. aL58ONBY was either analyzed in its “as isolated” state or after exposure to UV light (UVP BL-15; Analytik Jena US, Jena, Germany; CA 91786) for 20 min. Next, 100 pmol protein were injected and online buffer was exchanged to 200 mM ammonium acetate, pH 6.8 (AmAc) by a self-packed buffer exchange column [77] (P6 polyacrylamide gel, BioRad, Hercules, CA, USA) at a flow-rate of 100 µL per min. Mass spectra were recorded for 1000–8000 *m/z* at 35,000 resolution as defined at 200 *m/z*. The injection time was set to 200 ms. Voltages applied to the ion optics were optimized to allow for efficient ion transmission while minimizing unintentional ion activation. Only *m/z* corresponding to the monomer were considered for deconvolution and subsequent relative quantitation. Mass spectra were deconvoluted with UniDec version 4.0.0 beta [78] using the following processing parameters: Sample mass every 0.1 Da; peak FWHM 1 Thompson, Gaussian peak shape function.

### 4.11. CD Analysis

CD spectra in the far-UV range of 190–250 nm were recorded in a Jasco J-815 spectrophotometer (Jasco Deutschland, Pfungstadt, Germany) with five accumulations. The spectra were measured with 10–15 µM protein in 50 mM Tris HCl pH 7.5 in a 0.1 cm cuvette at 25 °C. Data were normalized to obtain the mean residue ellipticity as described in [79]. Thermal stabilities were recorded at 210 nm for 25−80 °C with a gradient of 1 °C per min. The curves were fitted with equation (5) in Origin 2018 (OriginLab, Northampton, MA, USA).

### 4.12. Analytical Size-Exclusion Chromatography

30 µM of TrpA monomer, TrpB monomer, or 30 µM TrpA mixed with 30 µM TrpB were subjected to a S75 10/300 GL (GE Healthcare, Chicago, IL, USA) column pre-equilibrated in 50 mM Tris·HCl pH 7.5. Likewise, 65 µM TrpA monomer, TrpB monomer, or 65 µM TrpA mixed with 65 µM TrpB were subjected to the same column pre-equilibrated in 50 mM Tris·HCl pH 7.5, 100 mM NaCl, 5 mM serine. Samples were eluted in the same buffer, and protein peaks were detected at 280 nm.

### 4.13. UV/Vis Spectral Analysis of Serine Binding

Absorbance titration experiments of covalent serine binding to TS were performed in a 1 cm quartz cuvette and an UV/Vis photometer (Jasco V750-UV/Vis spectrophotometer, Jasco Deutschland, Pfungstadt, Germany). Reduction of the internal aldimine peak signal at 412 nm was followed as a function of serine concentration. For this the spectra (350–700 nm) of 30 µM TS (1:1 TrpA:TrpB) in 50 mM, Tris·HCl pH 7.5, and alternatively 100 mM CsCl, were recorded after each step-wise addition of serine (0.001–15 mM end concentrations) and incubated at room temperature for 3 min. The internal aldimine peaks were isolated by linear baseline correction at wavelengths 380 nm and 450 nm, and the derived absorbance at 412 nm was subtracted from the initial absorbance at 0 mM serine. The resulting data were plotted against the serine concentration and fitted with equation (3) in Origin 2018 (OriginLab, Northampton, MA, USA) to obtain the apparent *K_d,app_* value of covalent serine binding.

### 4.14. Equations

Steady-state kinetics were evaluated by plotting the measured mean activity values *V* in [min^−1^] plus their standard error of mean (SEM) against the substrate concentration [S]. Michaelis-Menten constants *K_m_* and turnover numbers *k_cat_* were determined by fitting the data with the following equation [80]:

(1) $$V=\frac{k_{cat}\left[S\right]}{K_{m}+\left[S\right]}$$

In case of reactions that reach the same maximal velocity, such as the TrpB reaction in which either the serine or the indole concentration was kept in saturation, the experimental data of both reactions were fitted with a shared *k_cat_* in Origin 2018 (OriginLab, Northampton, MA, USA).

Substrate inhibition is a deviation from the usually hyperbolic Michalis-Menten behavior. Data were treated likewise, but fitted with the following equation to obtain *K_m_* and *k_cat_* values, as well as an inhibitory constant *K_i_* [80]:

(2) $$V=\frac{k_{cat}\left[S\right]}{K_{m}+\left[S\right]\left(1+\frac{\left[S\right]}{K_{i}}\right)}$$

Binding signals *y* were evaluated with the Hill equation to determine dissociation constants *K_d_*. Hill thereby considered cooperativity effects using a cooperativity coefficient *H*. If the start value (START) is unequal to zero, the following adaptation of the Hill equation of Origin 2018 (OriginLab, Northampton, MA, USA) can be used:

(3) $$y=START+\frac{\left(y_{max}-START\right){\left[x\right]}^{H}}{K_{d}{}^{H}+{\left[x\right]}^{H}}$$

For the evaluation of TrpA allosteric activation by wild-type TrpB, the mean activity values *y* were plotted against the wild-type TrpB concentration *x*, and the maximal velocity *y_max_* was determined with *H* = 2 representative of two TrpA monomers binding to one TrpB dimer. *K_d,app_^Ser^* values of covalent serine binding were determined by plotting the absorbance change *y* against the serine concentration *x*, and using *H* = 1 representative of one serine molecule binding to one TrpB active site.

Product inhibition was evaluated by normalizing the determined initial velocities (*V_i_*) to the initial velocity without indole (*V_0_*) and plotting them half-logarithmically against each indole concentration [I]. To identify IC50, the data were then fitted with the dose-response equation [81]:

(4) $$\frac{V_{i}}{V_{0}}=\frac{1}{1+\frac{\left[I\right]}{IC50}}$$

Thermal denaturation of proteins follows a Boltzmann-like behavior [82]. The CD signals (*y*) plotted against the respective temperatures *T* were hence fitted with the following Boltzmann equation with A1 and A2 as values of minimum and maximum intensities, respectively, to determine the melting point *T_m_*.

(5) $$y=\frac{A1-A2}{1+e^{\left(T-T_{m}\right)/dT}}+A2$$

## Figures and Tables

**Table 1:** Steady-state kinetics of TS and *ec*TS for the TrpA and TrpB partial reactions, and the overall TS reaction.

Reaction	Parameter	Dimension	Value TS	Value *ec*TS
TrpA ^1^	*k_cat_*	[min^−1^]	2.4 ± 0.2	1.5 ± 0.1
	*K_m_^IGP^*	[µM]	29.4 ± 8.4	17.6 ± 5.4
	*k_cat_*/*K_m_^IGP^*	[s^−1^M^−1^]	13.6 · 10^2^	14.2 · 10^2^
TrpB ^2^	*k_cat_*	[min^−1^]	24.1 ± 0.6	21.8 ± 0.7
	*K_m_^indole^*	[µM]	51.0 ± 4.5	10.6 ± 1.6
	*k_cat_*/*K_m_^indole^*	[s^−1^M^−1^]	7.9 · 10^3^	34.3 · 10^3^
	*K_m_^Ser^*	[mM]	0.53 ± 0.05	0.52 ± 0.07
	*k_cat_*/*K_m_^Ser^*	[s^−1^M^−1^]	7.6 · 10^2^	7.0 · 10^2^
TS ^3^	*k_cat_*	[min^−1^]	21.3 ± 0.6	28.3 ± 1.2
	*K_m_^IGP^*	[µM]	25.9 ± 2.6	24.9 ± 3.7
	*k_cat_*/*K_m_^IGP^*	[s^−1^M^−1^]	13.7 · 10^3^	18.9 · 10^3^
	*K_m_^Ser^*	[mM]	0.23 ± 0.03	0.35 ± 0.05
	*k_cat_*/*K_m_^Ser^*	[s^−1^M^−1^]	15.4 · 10^2^	13.5 · 10^2^

Experimental data are shown in Figure S2 and were fitted with the Michaelis-Menten equation (1). TrpB and TS reactions reach the same maximal velocity when either serine or indole/IGP is kept in saturation; corresponding data were consequently fitted with a shared *k_cat_* value. ^1^ IGP → GAP + indole. ^2^ Indole + Ser → Trp. ^3^ IGP + Ser → Trp.

**Table 2:** Steady-state kinetics of TS(aL58ONBY) in its “as isolated” state (ai) and after irradiation (hν), and TS(aL58Y) for the TrpA and TrpB partial reactions, and the overall TS reaction.

Reaction	Parameter	Dimension	Value TS(aL58ONBY^ai^)	LAF	Value TS(aL58ONBY^hν^)	Value TS(L58Y)
TrpA	***k_cat_***	[min^−1^]	0.19 ± 0.02	8 →	1.50 ± 0.07	2.30 ± 0.09
	***K_m_^IGP^***	[µM]	549 ± 92	3 ←	194 ± 23	234 ± 20
	***k_cat_*/*K_m_^IGP^***	[s^−1^M^−1^]	0.06 · 10^2^	**22 →**	1.29 · 10^2^	1.64 · 10^2^
TrpB	***k_cat_***	[min^−1^]	0.9 ± 0.0	13 →	12.1 ± 0.4	16.3 ± 0.3
	***K_m_^indole^***	[µM]	3.4 ± 0.4	4 →	13.9 ± 1.5	37.1 ± 2.4
	***k_cat_*/*K_m_^indole^***	[s^−1^M^−1^]	4.41 · 10^3^	**3 →**	14.51 · 10^3^	7.32 · 10^3^
	***K_m_^Ser^***	[mM]	3.9 ± 0.3	6 ←	0.7 ± 0.1	0.7 ± 0.0
	***k_cat_*/*K_m_^Ser^***	[s^−1^M^−1^]	0.04 · 10^2^	**72 →**	2.88 · 10^2^	3.88 · 10^2^
TS	***k_cat_***	[min^−1^]	0.3 ± 0.0	18 →	5.4 ± 0.2	10.9 ± 0.5
	***K_m_^IGP^***	[µM]	103 ± 18	2 ←	58 ± 10	29 ± 6
	***k_cat_*/*K_m_^IGP^***	[s^−1^M^−1^]	0.05 · 10^3^	**31 →**	1.55 · 10^3^	6.26 · 10^3^
	***K_m_^Ser^***	[mM]	0.6 ± 0.1	3 ←	0.2 ± 0.0	0.2 ± 0.0
	***k_cat_*/*K_m_^Ser^***	[s^−1^M^−1^]	0.08 · 10^2^	**56 →**	4.50 · 10^2^	9.08 · 10^2^

Experimental data are shown in Figure S4 and were fitted with the standard Michaelis-Menten equation (1) or a Michaelis-Menten equation taking into account substrate inhibition (2) in the case of the indole-dependent TrpB reaction. TrpB and TS reactions reached the same maximal velocity when either serine or indole/IGP was kept in saturation. Corresponding data were therefore fitted with a shared *k_cat_* value. LAF = light activation factor; the directions of value increase are indicated by arrows; the changes of the catalytic efficiencies are marked bold.

**Table 3:** Parameters for the stimulation of aL58ONBY^ai^ and aL58Y by wild-type TrpB in the absence and presence of serine. Experimental data are shown in Figure 5.

Reaction	Parameter	Dimension	Value TS(aL58ONBY)^ai^	Value TS(aL58Y)
TrpA (−Ser)	***V_0_^1^***	[µM min^−1^]	0.09 ± 0.01	0.16 ± 0.01
	***V_max_^2^***	[µM min^−1^]	0.12 ± 0.00	1.86 ± 0.06
	***V_max_*** */**V_0_**^3^*		1	12
TrpA (+Ser)	***V_0_***	[µM min^−1^]	0.02 ± 0.00	0.03 ± 0.04
	***V_max_***	[µM min^−1^]	0.09 ± 0.00	4.80 ± 0.33
	***V_max_*** */**V_0_***		4	160

^1^*V_0_* = mean initial velocity in absence of TrpB ± SEM of three independent measurements; ^2^ *V_max_* = maximal velocity in presence of TrpB ± SE as determined by curve fitting; ^3^ *V_max_/V_0_* = allosteric activation factor.

**Table 4:** Activity parameters of the direct photo-control of TS(aL58ONBY) compared to TS(aL58ONBY^ai^) and TS(aL58Y). Experimental data are shown in Figure 8.

Reaction	Parameter	Dimension	Value TS(aL58ONBY^ai^)	Value TS(aL58ONBY)	Value TS(aL58Y)
TrpA (−Ser)	***V_0_^1^***	[min^−1^]	0.15	0.17	1.27
	***V_hν_^2^***	[min^−1^]	—	0.73	0.84
	**LAF**		—	4	0.66
TrpA (+Ser)	***V_0_***	[min^−1^]	0.20	0.20	17.83
	***V_hν_***	[min^−1^]	—	8.46	10.01
	**LAF**		—	42	0.56
TS	***V_0_***	[min^−1^]	<0.03	<0.02	4.95
	***V_hν_***	[min^−1^]	—	2.83	3.10
	**LAF**		—	>141	0.63

^1^*V_0_* = initial velocity; *V_hν_* = velocity after irradiation; LAF = *V_hν_*/*V_0_*.

## Supplementary Materials

Supplementary Materials can be found at https://www.mdpi.com/1422-0067/20/20/5106/s1.

## Abbreviations

UAA	Unnatural amino acid
aaRS	Aminoacyl-tRNA synthetase
ONBY	*o*-nitrobenzyl-*O*-tyrosine
LAF	Light activation factor
TS	Tryptophan synthase from *Salmonella typhimurium*
IGP	Indole glycerol phosphate
GAP	Glyceraldehyde-3-phosphate
PLP	Pyridoxal phosphate
AA	Aminoacrylate
IA	Internal aldimine
TS	Tryptophan synthase from *Saccharomyces cerevisiae*
ecTS	Tryptophan synthase from *Escherichia coli*
HRP	Horse-radish peroxidase
MS	Mass spectrometry
MD	Molecular dynamics
CD	Circular dichroism
ai	“as isolated”
hν	Irradiated
SEM	Standard error of mean
SE	Standard error

## References

[1] Schmidt-Dannert C., Lopez-Gallego F. (2016). A roadmap for biocatalysis–functional and spatial orchestration of enzyme cascades. Microb. Biotechnol..

[2] Bornscheuer U.T., Höhne M. (2018). Protein Engineering: Methods and Protocols.

[3] Arnold F.H. (2018). Directed evolution: Bringing new chemistry to life. Angew. Chem. Int. Ed..

[4] Liu Q., Xun G., Feng Y. (2019). The state-of-the-art strategies of protein engineering for enzyme stabilization. Biotechnol. Adv..

[5] Woodley J.M. (2019). Accelerating the implementation of biocatalysis in industry. Appl. Microbiol. Biotechnol..

[6] Szymański W., Beierle J.M., Kistemaker H.A.V., Velema W.A., Feringa B.L. (2013). Reversible photocontrol of biological systems by the incorporation of molecular photoswitches. Chem. Rev..

[7] Schmermund L., Jurkaš V., Özgen F.F., Barone G.D., Büchsenschütz H.C., Winkler C.K., Schmidt S., Kourist R., Kroutil W. (2019). Photo-biocatalysis: Biotransformations in the presence of light. ACS Catal..

[8] Losi A., Gardner K.H., Möglich A. (2018). Blue-light receptors for optogenetics. Chem. Rev..

[9] Rost B.R., Schneider-Warme F., Schmitz D., Hegemann P. (2017). Optogenetic tools for subcellular applications in neuroscience. Neuron.

[10] Hüll K., Morstein J., Trauner D. (2018). In vivo photopharmacology. Chem. Rev..

[11] Baker A.S., Deiters A. (2014). Optical control of protein function through unnatural amino acid mutagenesis and other optogenetic approaches. ACS Chem. Biol..

[12] Bardhan A., Deiters A. (2019). Development of photolabile protecting groups and their application to the optochemical control of cell signaling. Curr. Opin. Struct. Biol..

[13] Liu C.C., Schultz P.G. (2010). Adding new chemistries to the genetic code. Annu. Rev. Biochem..

[14] Deiters A., Groff D., Ryu Y., Xie J., Schultz P.G. (2006). A genetically encoded photocaged tyrosine. Angew. Chem. Int. Ed..

[15] Chou C., Young D.D., Deiters A. (2009). A light-activated DNA polymerase. Angew. Chem. Int. Ed..

[16] Chou C., Young D.D., Deiters A. (2010). Photocaged T7 RNA polymerase for the light activation of transcription and gene function in pro- and eukaryotic cells. ChemBioChem.

[17] Wang J., Liu Y., Liu Y., Zheng S., Wang X., Zhao J., Yang F., Zhang G., Wang C., Chen P.R. (2019). Time-resolved protein activation by proximal decaging in living systems. Nature.

[18] Luo J., Torres-Kolbus J., Liu J., Deiters A. (2017). Genetic encoding of photocaged tyrosines with improved light-activation properties for the optical control of protease function. ChemBioChem.

[19] Larson A.S., Hergenrother P.J. (2014). Light activation of *Staphylococcus aureus* toxin YoeBSa1 reveals guanosine-specific endoribonuclease activity. Biochemistry.

[20] Zhang G., Li J., Xie R., Fan X., Liu Y., Zheng S., Ge Y., Chen P.R. (2016). Bioorthogonal chemical activation of kinases in living systems. ACS Cent. Sci..

[21] Motlagh H.N., Wrabl J.O., Li J., Hilser V.J. (2014). The ensemble nature of allostery. Nature.

[22] Kastritis P.L., Gavin A.-C. (2018). Enzymatic complexes across scales. Essays Biochem..

[23] Makhlynets O.V., Raymond E.A., Korendovych I.V. (2015). Design of allosterically regulated protein catalysts. Biochemistry.

[24] Romney D.K., Murciano-Calles J., Wehrmüller J.E., Arnold F.H. (2017). Unlocking reactivity of TrpB: A general biocatalytic platform for synthesis of tryptophan analogues. J. Am. Chem. Soc..

[25] Miles E.W. (1995). Tryptophan synthase. Structure, function, and protein engineering. Subcell. Biochem..

[26] Raboni S., Bettati S., Mozzarelli A. (2009). Tryptophan synthase: A mine for enzymologists. Cell. Mol. Life Sci..

[27] Barends T.R.M., Dunn M.F., Schlichting I. (2008). Tryptophan synthase, an allosteric molecular factory. Curr. Opin. Chem. Biol..

[28] Weyand M., Schlichting I. (1999). Crystal structure of wild-type tryptophan synthase complexed with the natural substrate indole-3-glycerol phosphate. Biochemistry.

[29] Hyde C.C., Ahmed S.A., Padlan E.A., Miles E.W., Davies D.R. (1988). Three-dimensional structure of the tryptophan synthase α_2_β_2_ multienzyme complex from *Salmonella typhimurium*. J. Biol. Chem..

[30] Dunn M.F. (2012). Allosteric regulation of substrate channeling and catalysis in the tryptophan synthase bienzyme complex. Arch. Biochem. Biophys..

[31] Mozzarelli A., Peracchi A., Rossi G.L., Ahmed S.A., Miles E.W. (1989). Microspectrophotometric studies on single crystals of the tryptophan synthase alpha 2 beta 2 complex demonstrate formation of enzyme-substrate intermediates. J. Biol. Chem..

[32] Dunn M.F., Aguilar V., Brzovic P., Drewe W.F., Houben K.F., Leja C.A., Roy M. (1990). The tryptophan synthase bienzyme complex transfers indole between the α- and β-sites via a 25–30 Å long tunnel. Biochemistry.

[33] Anderson K.S., Miles E.W., Johnson K.A. (1991). Serine modulates substrate channeling in tryptophan synthase. A novel intersubunit triggering mechanism. J. Biol. Chem..

[34] Miles E.W. (1991). The tryptophan synthase α_2_β_2_ complex: Cleavage of a flexible loop in the α subunit alters allosteric properties. J. Biol. Chem..

[35] Peracchi A., Mozzarelli A., Rossi G.L. (1995). Monovalent cations affect dynamic and functional properties of the tryptophan synthase α_2_β_2_ complex. Biochemistry.

[36] Woehl E., Dunn M.F. (1999). Mechanisms of monovalent cation action in enzyme catalysis:  The tryptophan synthase α-, β-, and αβ-reactions. Biochemistry.

[37] Miles E.W. (1979). Tryptophan synthase: Structure, function, and subunit interaction. Adv. Enzymol. Relat. Areas Mol. Biol..

[38] Buller A.R., Brinkmann-Chen S., Romney D.K., Herger M., Murciano-Calles J., Arnold F.H. (2015). Directed evolution of the tryptophan synthase β-subunit for stand-alone function recapitulates allosteric activation. Proc. Natl. Acad. Sci. USA.

[39] Buller A.R., van Roye P., Cahn J.K.B., Scheele R.A., Herger M., Arnold F.H. (2018). Directed evolution mimics allosteric activation by stepwise tuning of the conformational ensemble. J. Am. Chem. Soc..

[40] Zhang H., Wang Q., Ning X., Hang H., Ma J., Yang X., Lu X., Zhang J., Li Y., Niu C. (2015). Synthesis and biological evaluations of a series of thaxtomin analogues. J. Agric. Food Chem..

[41] Zhang H., Ning X., Hang H., Ru X., Li H., Li Y., Wang L., Zhang X., Yu S., Qiao Y. (2013). Total synthesis of thaxtomin A and its stereoisomers and findings of their biological activities. Org. Lett..

[42] Boville C.E., Scheele R.A., Koch P., Brinkmann-Chen S., Buller A.R., Arnold F.H. (2018). Engineered biosynthesis of β-alkyl tryptophan analogues. Angew. Chem. Int. Ed..

[43] Francis D., Winn M., Latham J., Greaney M.F., Micklefield J. (2017). An engineered tryptophan synthase opens new enzymatic pathways to β-methyltryptophan and derivatives. ChemBioChem.

[44] Dick M., Sarai N.S., Martynowycz M.W., Gonen T., Arnold F.H. (2019). Tailoring tryptophan synthase TrpB for selective quaternary carbon bond formation. ChemRxiv.

[45] Creighton T.E. (1970). A steady-state kinetic investigation of the reaction mechanism of the tryptophan synthetase of *Escherichia coli*. Eur. J. Biochem..

[46] Lane A.N., Kirschner K. (1983). The catalytic mechanism of tryptophan synthase from *Escherichia coli*. Eur. J. Biochem..

[47] Wellington S., Nag P.P., Michalska K., Johnston S.E., Jedrzejczak R.P., Kaushik V.K., Clatworthy A.E., Siddiqi N., McCarren P., Bajrami B. (2017). A small-molecule allosteric inhibitor of *Mycobacterium tuberculosis* tryptophan synthase. Nat. Chem. Biol..

[48] Kameya M., Onaka H., Asano Y. (2013). Selective tryptophan determination using tryptophan oxidases involved in bis-indole antibiotic biosynthesis. Anal. Biochem..

[49] Lane A.N., Kirschner K. (1991). Mechanism of the physiological reaction catalyzed by tryptophan synthase from *Escherichia coli*. Biochemistry.

[50] Kirschner K., Lane A.N., Strasser A.W.M. (1991). Reciprocal communication between the lyase and synthase active sites of the tryptophan synthase bienzyme complex. Biochemistry.

[51] McGaughey G.B., Gagné M., Rappé A.K. (1998). π-Stacking Interactions: Alive and well in proteins. J. Biol. Chem..

[52] Schneider T.R., Gerhardt E., Lee M., Liang P.-H., Anderson K.S., Schlichting I. (1998). Loop closure and intersubunit communication in tryptophan synthase. Biochemistry.

[53] Rhee S., Parris K.D., Ahmed S.A., Miles E.W., Davies D.R. (1996). Exchange of K^+^ or Cs^+^ for Na^+^ induces local and long-range changes in the three-dimensional structure of the tryptophan synthase α2β2 complex. Biochemistry.

[54] Il’ichev Y.V., Wirz J. (2000). Rearrangements of 2-nitrobenzyl compounds: 1. Potential energy surface of 2-nitrotoluene and its isomers explored with ab initio and density functional theory methods. J. Phys. Chem. A.

[55] Böcker J.K., Dörner W., Mootz H.D. (2019). Light-control of the ultra-fast Gp41-1 split intein with preserved stability of a genetically encoded photo-caged amino acid in bacterial cells. Chem. Commun..

[56] Ruvinov S.B., Yang X.-J., Parris K.D., Banik U., Ahmed S.A., Miles E.W., Sackett D.L. (1995). Ligand-mediated changes in the tryptophan synthase indole tunnel probed by nile red fluorescence with wild type, mutant, and chemically modified enzymes. J. Biol. Chem..

[57] Anderson K.S., Kim A.Y., Quillen J.M., Sayers E., Yang X.-J., Miles E.W. (1995). Kinetic characterization of channel impaired mutants of tryptophan synthase. J. Biol. Chem..

[58] Weyand M., Schlichting I. (2000). Structural basis for the impaired channeling and allosteric inter-subunit communication in the βA169L/βC170W mutant of tryptophan synthase. J. Biol. Chem..

[59] Harris R.M., Dunn M.F. (2002). Intermediate trapping via a conformational switch in the Na^+^-activated tryptophan synthase bienzyme complex. Biochemistry.

[60] Abrahams K.A., Cox J.A.G., Fütterer K., Rullas J., Ortega-Muro F., Loman N.J., Moynihan P.J., Pérez-Herrán E., Jiménez E., Esquivias J. (2017). Inhibiting mycobacterial tryptophan synthase by targeting the inter-subunit interface. Sci. Rep..

[61] Kneuttinger A.C., Straub K., Bittner P., Simeth N.A., Bruckmann A., Busch F., Rajendran C., Hupfeld E., Wysocki V.H., Horinek D. (2019). Light regulation of enzyme allostery through photo-responsive unnatural amino acids. Cell Chem. Biol..

[62] Slanina T., Shrestha P., Palao E., Kand D., Peterson J.A., Dutton A.S., Rubinstein N., Weinstain R., Winter A.H., Klán P. (2017). In search of the perfect photocage: Structure–reactivity relationships in meso-methyl BODIPY photoremovable protecting groups. J. Am. Chem. Soc..

[63] Walton D.P., Dougherty D.A. (2017). A general strategy for visible-light decaging based on the quinone trimethyl lock. J. Am. Chem. Soc..

[64] Bose M., Groff D., Xie J., Brustad E., Schultz P.G. (2006). The incorporation of a photoisomerizable amino acid into proteins in *E. coli*. J. Am. Chem. Soc..

[65] Luo J., Samanta S., Convertino M., Dokholyan N.V., Deiters A. (2018). Reversible and tunable photoswitching of protein function through genetic encoding of azobenzene amino acids in mammalian cells. ChemBioChem.

[66] John A.A., Ramil C.P., Tian Y., Cheng G., Lin Q. (2015). Synthesis and site-specific incorporation of red-shifted azobenzene amino acids into proteins. Org. Lett..

[67] Busch F., Rajendran C., Mayans O., Löffler P., Merkl R., Sterner R. (2014). TrpB2 enzymes are *O*-phospho-L-serine dependent tryptophan synthases. Biochemistry.

[68] Leopoldseder S., Hettwer S., Sterner R. (2006). Evolution of multi-enzyme complexes: The case of tryptophan synthase. Biochemistry.

[69] Young T.S., Ahmad I., Yin J.A., Schultz P.G. (2010). An enhanced system for unnatural amino acid mutagenesis in *E. coli*. J. Mol. Biol..

[70] Schlee S., Dietrich S., Kurćon T., Delaney P., Goodey N.M., Sterner R. (2013). Kinetic mechanism of indole-3-glycerol phosphate synthase. Biochemistry.

[71] Rohweder B., Semmelmann F., Endres C., Sterner R. (2018). Standardized cloning vectors for protein production and generation of large gene libraries in *Escherichia coli*. BioTechniques.

[72] Engler C., Kandzia R., Marillonnet S. (2008). A one pot, one step, precision cloning method with high throughput capability. PLoS ONE.

[73] Krieger E., Darden T., Nabuurs S.B., Finkelstein A., Vriend G. (2004). Making optimal use of empirical energy functions: Force-field parameterization in crystal space. Proteins.

[74] Mitchell A., Chang H.-Y., Daugherty L., Fraser M., Hunter S., Lopez R., McAnulla C., McMenamin C., Nuka G., Pesseat S. (2014). The InterPro protein families database: The classification resource after 15 years. Nucleic Acids Res..

[75] Crooks G.E., Hon G., Chandonia J.-M., Brenner S.E. (2004). WebLogo: A sequence logo generator. Genome Res..

[76] VanAernum Z.L., Gilbert J.D., Belov M.E., Makarov A.A., Horning S.R., Wysocki V.H. (2019). Surface-induced dissociation of noncovalent protein complexes in an extended mass range orbitrap mass spectrometer. Anal. Chem..

[77] VanAernum Z., Busch F., Jones B.J., Jia M., Chen Z., Boyken S.E., Sahasrabuddhe A., Baker D., Wysocki V. (2019). Rapid online buffer exchange: A method for screening of proteins, protein complexes, and cell lysates by native mass spectrometry. ChemRxiv.

[78] Marty M.T., Baldwin A.J., Marklund E.G., Hochberg T.K.A., Benesch J.L.P., Robinson C.V. (2015). Bayesian deconvolution of mass and ion mobility spectra: from binary interactions to polydisperse ensembles. Anal. Chem..

[79] Kelly S.M., Jess T.J., Price N.C. (2005). How to study proteins by circular dichroism. Biochim. Biophys. Acta.

[80] Copeland R.A., Copeland R.A. (2002). Kinetics of single-substrate enzyme reactions. Enzymes.

[81] Copeland R.A., Copeland R.A. (2002). Reversible inhibitors. Enzymes.

[82] Finkelstein A.V., Badretdinov A.Y., Gutin A.M. (1995). Why do protein architectures have boltzmann-like statistics?. Proteins.

