# Supplementary material for: Light-Regulation of Tryptophan Synthase by Combining Protein Design and Enzymology
Source: Int J Mol Sci. 2019 Oct 15;20(20):5106. doi: 10.3390/ijms20205106 (PMC6829457; doi:10.3390/ijms20205106)
Supplement: Supplementary file 1 [file ijms-20-05106-s001.pdf]

International Journal of *Molecular Sciences*

MDPI

Supplementary Information

Light-Regulation of Tryptophan Synthase by Combining Protein Design and Enzymology

Andrea C. Kneuttinger [1], Stefanie Zwisele [1], Kristina Straub [1], Astrid Bruckmann [2], Florian Busch [3], Thomas Kinateder [1], Barbara Gaim [1], Vicki H. Wysocki [3], Rainer Merkl [1], and Reinhard Sterner [1,*]

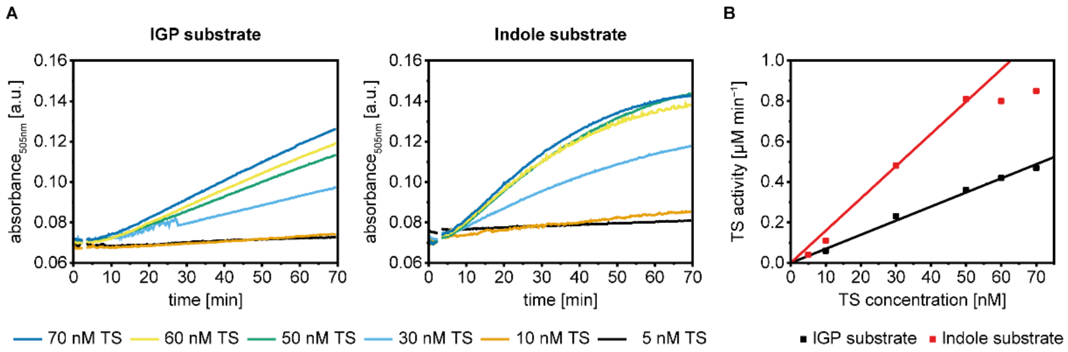

Figure S1. Establishment of a continuous assay for the detection of tryptophan in steady-state kinetics. **(A)** The production of tryptophan from IGP or indole with serine, respectively, is followed spectrophotometrically at 505 nm over time by coupling the reaction to the tryptophan oxidase VioA and the peroxidase HRP resulting in colored quinoneimine. **(B)** The determined initial catalytic rate for tryptophan formation is linearly dependent on TS concentration proofing that TS is rate-limiting and that the assay is well-suited for steady-state kinetics with a TS concentration of up to ~50 nM. Reaction conditions: 50 mM KP pH 7.5, 90 mM KCl, 1 g/L VioA, 0.15 g/L HRP, 5 mM serine, 170 µM indole/IGP, 20 µM PLP, 1 mM phenol, 1 mM 4-aminoantipyrin, 5–70 nM TS (1:1 TrpA:TrpB) at 25 °C.

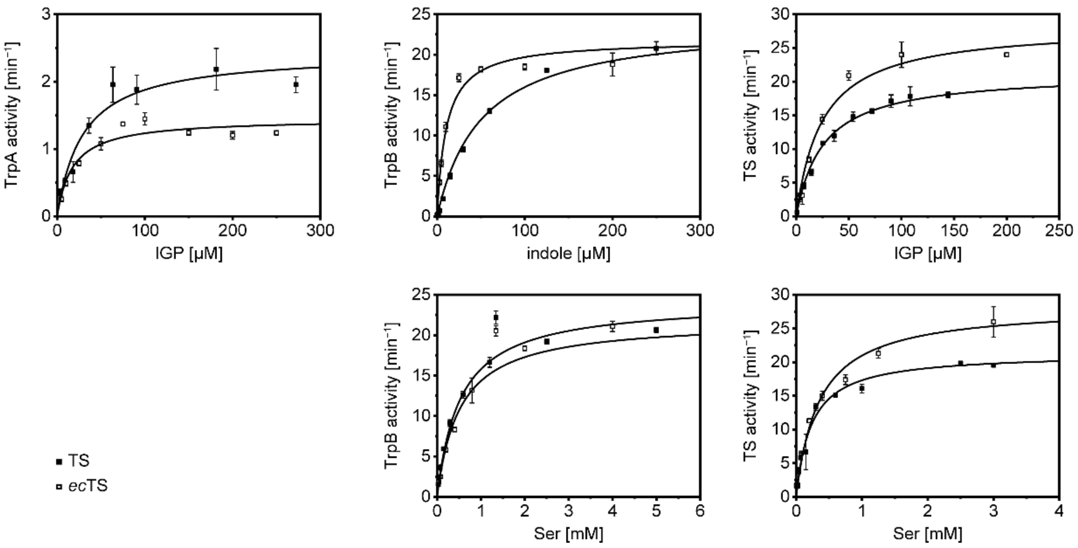

Figure S2. Steady-state kinetics of wt-TS and wt-*ec*TS for the TrpA (left panel) and TrpB (middle panels) partial reactions, and the overall TS reaction (right panels). Reaction conditions of TrpA activity: 50 mM Tris·HCl pH 7.8, 100 mM NaCl, 5 µM GAP dehydrogenase, 5–250 µM IGP, 5 mM NAD^+, 20 mM Na_2HAsO_4, 40 µM PLP, and 1 µM TS (1:1 TrpA:TrpB). Reaction conditions of TrpB and TS activity: 50 mM KP pH 7.5, 100 mM KCl, 1 g/L VioA, 0.15 g/L HRP, 0.015–5 mM (or 5 mM for saturation) serine, 1–250 µM (or 500 µM for saturation) indole / 3–250 µM (or 500 µM for saturation) IGP, and 20 nM TS (1:1 TrpA:TrpB). All reactions were performed in triplicates at 25 °C (data shown ± SEM) and data were fitted with the Michaelis-Menten equation (1). TrpB and TS reactions reach the same maximal velocity when either serine or indole/IGP is kept in saturation; that is why the corresponding curves were fitted with a shared k_{cat} value.

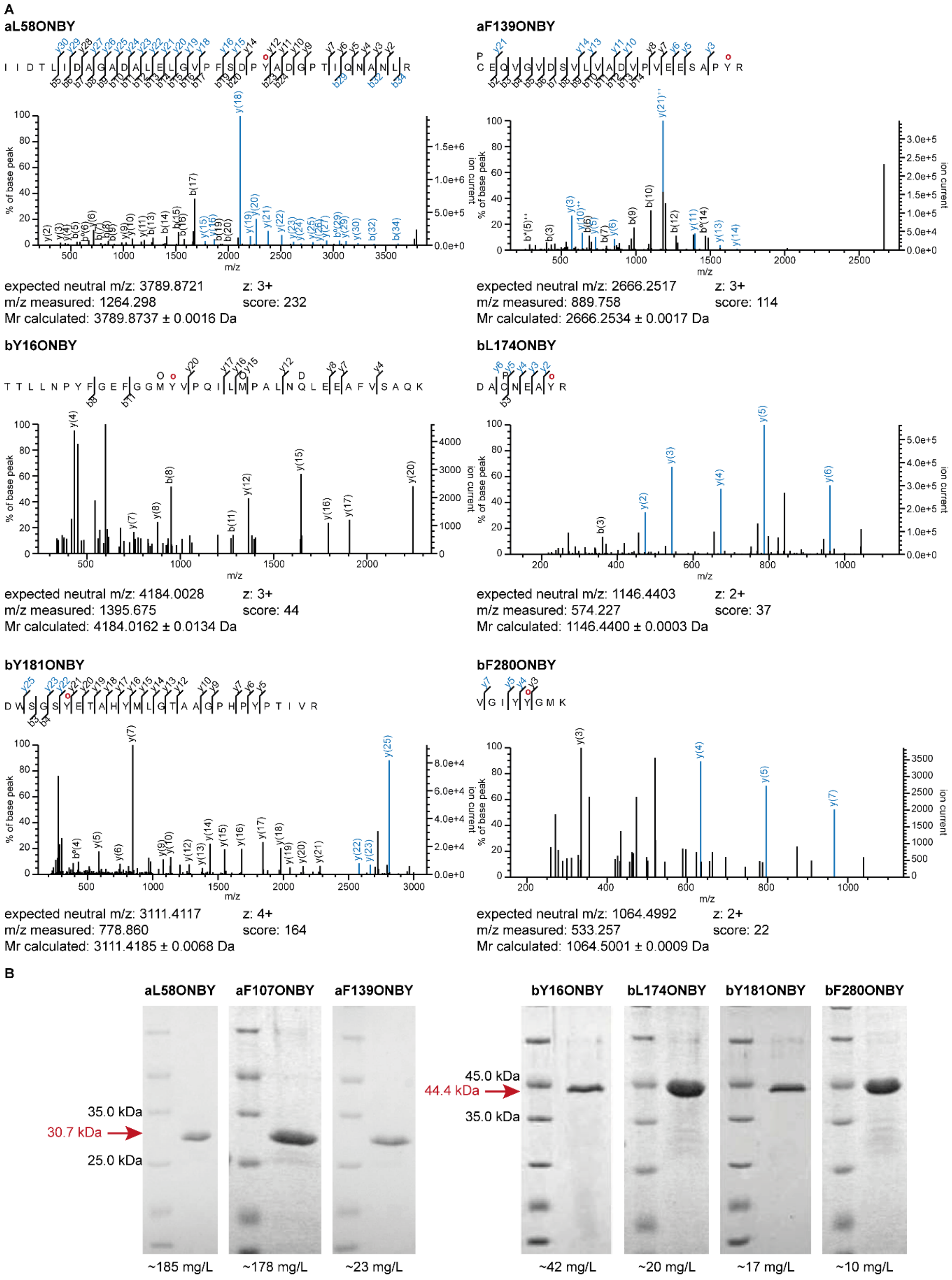

Figure S3. Confirmed identity and purity of ONBY-TrpAs and ONBY-TrpBs. **(A)** For each ONBY-TrpA and ONBY-TrpB, except aF107ONBY, the fragment spectrum of the tryptic digest coupled to liquid mass spectrometry (LC-MS) is shown. Above the fragment spectrum, the semi-tryptic peptide harboring ONBY gives an overview of all measured fragments; fragments directly containing ONBY are highlighted in blue. **o**: ONBY, D: deamidated, O: oxidated, P: propionamidated. **(B)** SDS-PAGE of all ONBY-TrpA and ONBY-TrpB proteins (3 µg), demonstrating that the proteins are >90% pure. The purification yield (protein per liter expression culture) is shown below the SDS gels.

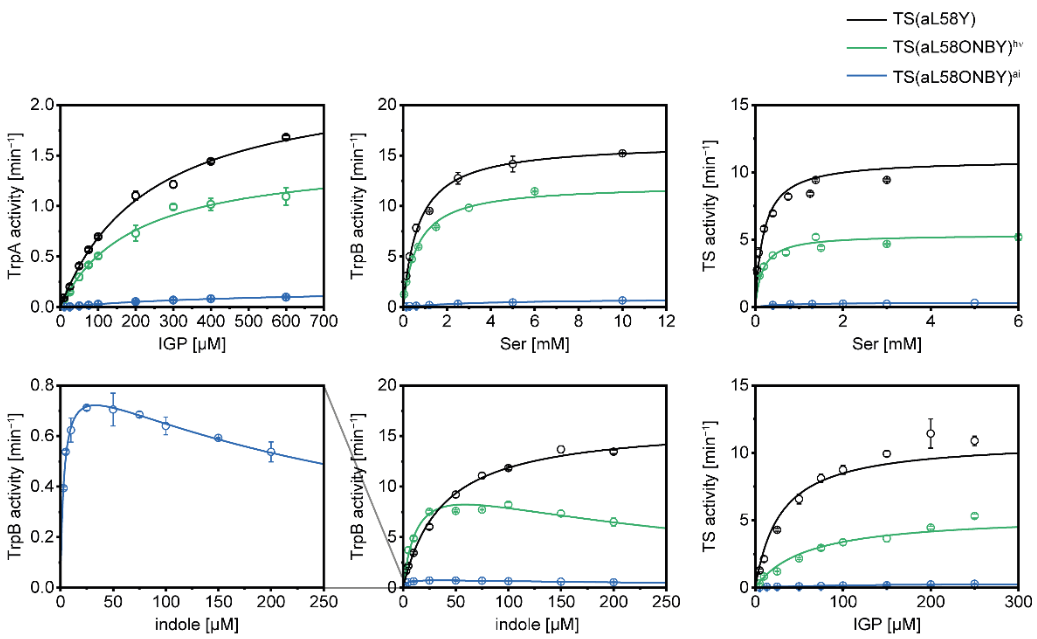

Figure S4. Steady-state kinetics of TS(aL58ONBY) in its "as isolated" (ai) and irradiated (hν) state and TS(aL58Y) for the TrpA (upper left panel) and TrpB (middle and lower left panels) partial reactions, and the overall TS reaction (right panels). Reaction conditions for TrpA activity: 50 mM Tris·HCl pH 7.8, 100 mM NaCl, 5 µM GAP dehydrogenase, 10–250 µM IGP, 5 mM NAD^+, 20 mM Na_2HAsO_4, 40 µM PLP, and 1 µM (TS(aL58ONBY)$^{h\nu}$ and TS(aL58Y)) or 5 µM (TS(aL58ONBY)^{ai}) TS (1:1 TrpA:TrpB). Reaction conditions for TrpB and TS activities: 50 mM KP pH 7.5, 100 mM KCl, 1 g/L VioA, 0.15 g/L HRP, 0.015–10 mM (or 5 mM for saturation) serine, 3–200 µM (or 500 µM for saturation) indole / 5–250 µM (or 500 µM for saturation) IGP, 40 µM PLP, 1 mM phenol, 1 mM 4-aminoantipyrin, 20 nM (TS(aL58ONBY)$^{h\nu}$ and TS(aL58Y)) or 1 µM (TS(aL58ONBY)^{ai}) TS (1:1 TrpA:TrpB). All reactions were performed in triplicates at 25 °C (data shown ± SEM) and data were fitted with the Michaelis-Menten (1) or substrate inhibition (2) equations, respectively. TrpB and TS reactions reach the same maximal velocity when either serine or indole/IGP is kept in saturation; that is why the corresponding curves were fitted with a shared k_{cat} value.
